# Models of Molecular Structures of Hexa-Nuclear Al_n_Fe_m_ Metal Clusters (n + m = 6): DFT Quantum-Chemical Design

**DOI:** 10.3390/ma14030597

**Published:** 2021-01-27

**Authors:** Oleg V. Mikhailov, Denis V. Chachkov

**Affiliations:** 1 Department of Analytical Chemistry, Certification and Quality Management, Kazan National Research Technological University, K. Marx Street 68, 420015 Kazan, Russia; 2 Kazan Department of Joint Supercomputer Center of Russian Academy of Sciences—Branch of Federal Scientific Center “Scientific Research Institute for System Analysis of the RAS”, Lobachevskii Street 2/31, 420111 Kazan, Russia; de2005c@gmail.com

**Keywords:** metal cluster, aluminum, iron, molecular structure, DFT method

## Abstract

By using the density functional theory (DFT) method at the OPBE/QZVP level, key parameters of molecular structures of six-atomic (heterobi)nuclear metal clusters with an Al_n_Fe_m_ composition (n + m = 6) (bond lengths, bond angles, and torsion (dihedral) angles) were calculated. It was found that each of these clusters exists in a large number of structural isomers that differ substantially in terms of their total energy. Furthermore, the molecular structures of these structural isomers significantly differ regarding the geometric parameters and geometric form. In addition, the most stable structural isomers of these metal clusters also differ rather considerably in terms of the geometric form.

## 1. Introduction

In previous studies [1,2,3,4,5,6,7,8,9,10,11,12,13,14,15,16,17,18,19,20,21,22,23,24,25,26,27,28,29,30,31,32,33], quantum chemical calculations of metal clusters containing atoms of two variable *p-* and *d*-elements, known as (*dd*)- and (*pd*)heterobimetallic metal clusters, were carried out using the density functional theory (DFT) method. In the works [1,2,3,4,5,6,7,8,9,10,11,12,13,14], the objects of study were (*dd*)heterobimetallic metal clusters which included atoms of two different *d*-elements, in particular, (Cu, Fe) [1], (Pd, Fe) [2], (Pd, Ag) [3,4,5], (Pt, Cu) [6], (Au, Fe) [7], (Au, Pd) [8], (Au, Ag) [9], (Au, Ir) [12], and (Pd, Ir) [14]. Some of these metal clusters have been applied in various fields of science and technology [10,11,12,13,14]. The *(pd*)heterobimetallic metal clusters that include atoms of different metal categories, namely, *p*- and *d*-elements, are of greater interest than (*dd*)heterobimetallic ones, because, theoretically, it can be expected that they will demonstrate new properties that are not inherent to metal clusters containing metal atoms of only one category of chemical elements.

Among the most important *p*-elements is aluminum, which has a very wide industrial application; nevertheless, only a few (*pd*)metal clusters containing this *p*-element and any of the *d*-metals are described in the literature [15,16,17,18,19,20,21,22,23,24,25,26,27,28,29,30,31,32,33]. On the other hand, the most important *d*-element is iron, which plays a key role in the iron and steel industry. Metal clusters containing aluminum and iron are of undoubted interest, if only because they are structural units of bimetallic alloys such as “alfer” and “alphenol”, containing 13% and 16% aluminum, respectively, and having a high magnetic permeability in weak magnetic fields and significant electrical resistance. In addition, both of them have a fairly high hardness, strength, and wear resistance, due to which the first of these alloys is used for the manufacturing of electroacoustic (magneto-strictive) transducers, and the second, in the production of cores for recording and reproducing heads of magnetic recording equipment. Based on the specifics of the phase diagram of the (aluminum–iron) system and the composition of the (AlFe) intermetallic compounds formed within it, it may be assumed that the key structural units of these compounds are tetra-, penta-, and hexa-nuclear (AlFe) metal clusters. However, quantum-chemical calculations (AlFe) of metal clusters containing four to six atoms have only been performed in a few works [23,24,25,26,27,28,29,30,31,32,33], and in all cases, the DFT method with the OPBE/TZVP basis set was used. In our opinion, hexa-nuclear aluminum–iron metal clusters with the general formula Al_n_Fe_m_, where the values of n and m vary from 1 to 5, are of the greatest interest here. Considering this, this article will be devoted to the presentation and systematization of the quantum-chemical calculation results of such compounds’ molecular structures using a more advanced method in the framework of density functional theory (DFT), specifically DFT OPBE/QZVP.

## 2. Results

**Figure 1:**
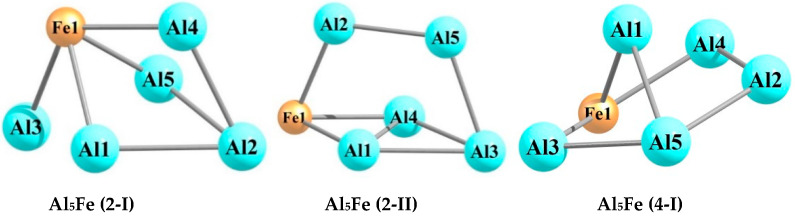

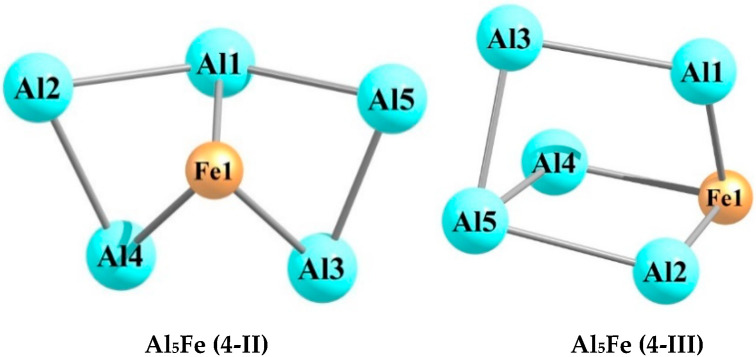
Molecular structures of the five most energetically stable Al_5_Fe metal clusters.

**Figure 2:**
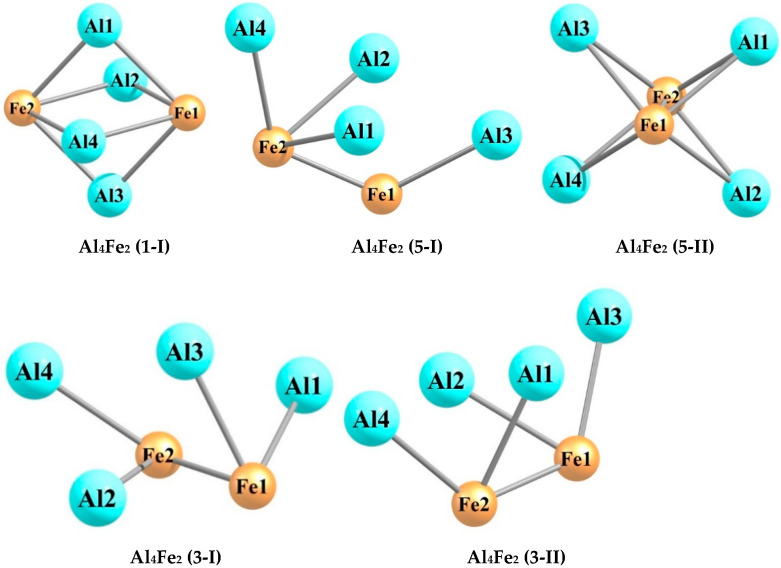
Molecular structures of the five most energetically stable Al_4_Fe_2_ metal clusters.

**Figure 3:**
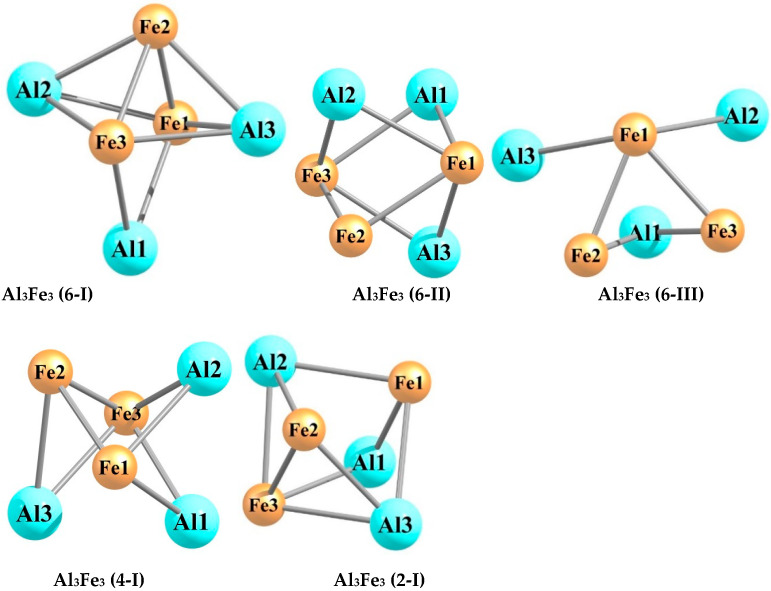
Molecular structures of the five most energetically stable Al_3_Fe_3_ metal clusters.

**Figure 4:**
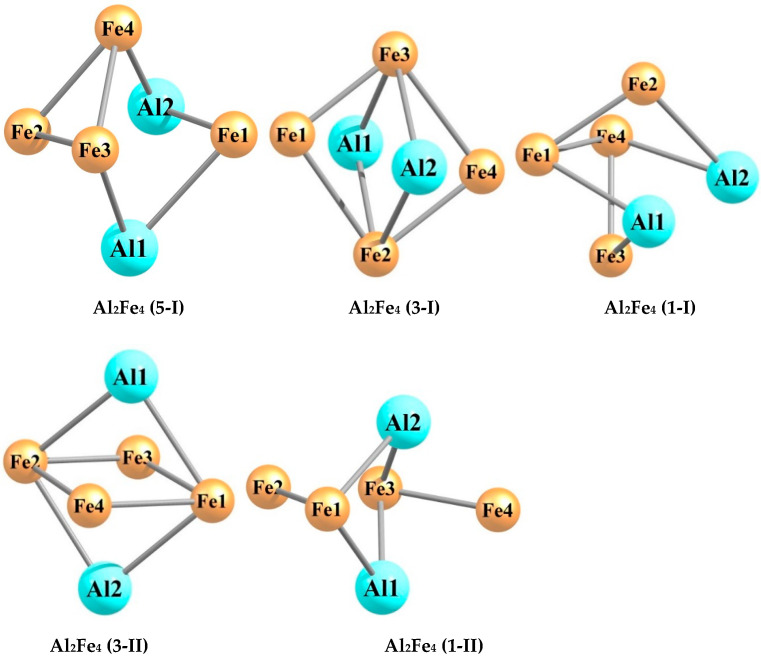
Molecular structures of the five most energetically stable Al_2_Fe_4_ metal clusters.

**Figure 5:**
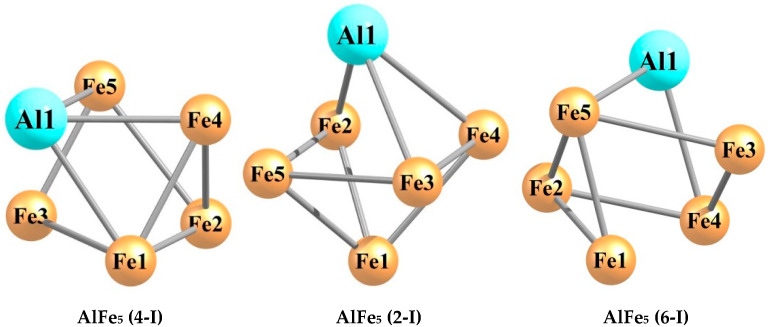

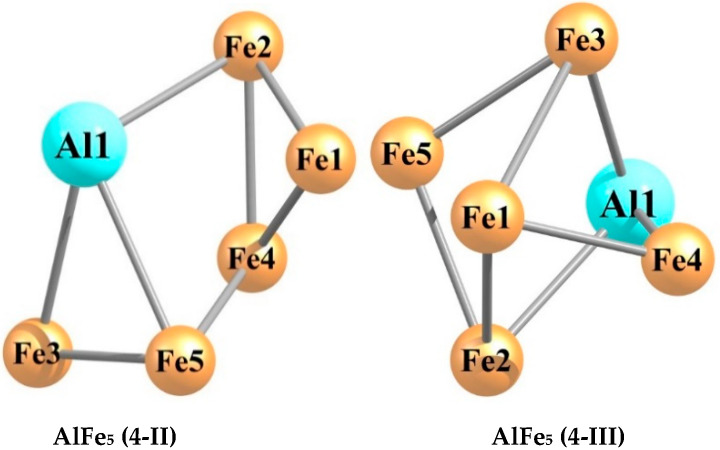
Molecular structures of the five most energetically stable AlFe_5_ metal clusters.

The quantum chemical calculations carried out by using the DFT method at the OPBE/QZVP level showed that each of the hexa-nuclear aluminum–iron metal clusters with the stoichiometric composition Al_n_Fe_m_ (n + m = 6), namely, Al_5_Fe, Al_4_Fe_2_, Al_3_Fe_3_, Al_2_Fe_4_, and AlFe_5_, exists in a very significant number of structural isomers, the number of which varies from 19 in the case of Al_5_Fe to 40 in the case of Al_3_Fe_3_. The information of these metal clusters in detail is presented in the Table 1, Table 2, Table 3, Table 4, Table 5, Table 6, Table 7, Table 8, Table 9 and Table 10. As may be seen from the Table 1, Table 3, Table 5, Table 7, and Table 9, the relative total energies of these structural isomers for each of the metal clusters under examination also vary in a very wide range. The most energetically advantageous among these isomers for Al_5_Fe, Al_4_Fe_2_, Al_3_Fe_3_, Al_2_Fe_4_, and AlFe_5_ are shown in Figure 1, Figure 2, Figure 3, Figure 4 and Figure 5. The molecular structure parameters of these structural isomers are given in Table 2, Table 4, Table 6, Table 8, and Table 10, respectively. A complete assortment of molecular structures of all these metal clusters is presented in the *Supplemental Materials*. In terms of the numbering of metal clusters, the Arabic numeral in parentheses denotes the value of the spin multiplicity of the ground state (*M_S_*), and the Roman numeral is the ordinal number of the metal cluster with *M_S_* data in ascending relative energy.

NBO analysis data for the metal clusters indicated in Table 11 are presented in Table 12.

## 3. Discussion

As can be seen from the presented data, the Al_5_Fe metal cluster exists in 19 structural isomers, the Al_4_Fe_2_ metal cluster in 39 structural isomers, the Al_3_Fe_3_ metal cluster in 40 structural isomers, the Al_2_Fe_4_ metal cluster in 53 structural isomers, and the AlFe_5_ metal cluster in 23 structural isomers. There is no regularity in these numbers; there is only a tendency for them to first increase their number with an increase in the number of iron atoms (m) in the structural unit of the metal cluster (in the range m = 1–4), and then to decrease (in the range m = 4–5). For any of the considered metal clusters in the series Al_5_Fe–Al_4_Fe_2_–Al_3_Fe_3_–Al_2_Fe_4_–AlFe_5_, in general, a very significant variety of molecular structures is characteristic–from pseudo-octahedral [Al_2_Fe_4_ (3-V)] to strictly planar [Al_4_Fe_2_ (3-XIII)]. Both of these structures are unique and are not observed in any of the other Al_n_Fe_m_ metal clusters (n + m = 6). For the overwhelming majority of the presented metal cluster structures, a pronounced asymmetry with an almost complete absence of any symmetrical elements is typical. Therefore, none of these metal clusters has a symmetry axis of the third or higher order, and only some of them have a plane of symmetry and a symmetry center (see Supplementary Materials). This fully applies to the most energetically stable (i.e., those with the minimum total energy values) metal clusters presented in Figure 1, Figure 2, Figure 3, Figure 4 and Figure 5. It is important to note that in pairs of so-called “inverted” metal clusters, where the numerical values of m and n for Al and Fe atoms are interchanged [(Al_5_Fe, AlFe_5_) and (Al_4_Fe_2_, Al_2_Fe_4_)], there is no noticeable similarity between the assortments of molecular structures in either quantitative or qualitative relations.

A remarkable feature of all the Al_n_Fe_m_ metal clusters is the relatively small number of metal–metal bonds—no more than 8; at the same time, there is no apparent correlation between the total number of these bonds in the metal cluster and its energy stability (i.e., the value of its relative energy). It is very curious that the formation of Al–Al bonds in molecular structures of these metal clusters is much rarer than the formation of Fe–Fe bonds, and this phenomenon even takes place in the case of the Al_4_Fe_2_ metal cluster, where the number of aluminum atoms in each of the 39 molecular structures is twice as high as the number of iron atoms (in the most energetically stable Al_4_Fe_2_ metal cluster, namely Al_4_Fe_2_ (1-I), there is also no Al–Al bond (Figure 2)). Among the molecular structures of the Al_2_Fe_4_ metal cluster, there is not even one structure in which such a bond is present; the most energetically stable of them—Al_2_Fe_4_ (5-I)—is no exception (Figure 4). Moreover, Figure 2, Figure 3 and Figure 4 show that, among the five most stable structural isomers of each of the metal clusters Al_2_Fe_4_, Al_3_Fe_3_, and Al_2_Fe_4_, there is not a single one in which the molecular structure contains at least one Al–Al bond. Molecular structures with the absence of Fe–Fe bonds only occur in the case of the Al_5_Fe metal cluster (where such a bond cannot exist in principle) and the Al_4_Fe_2_ metal cluster, so they are in the minority compared to structures where the Fe–Fe bond is present (16 out of 39 molecular structures). Al–Fe bonds, as was expected, are present in each of the structural isomers of each metal cluster under consideration. It should be noted that the length of any of the metal–metal bonds in any of these metal clusters is more than 200 pm (see Table 2, Table 4, Table 6, Table 8, and Table 10). The Al–Al bonds are the longest on average, the Fe–Fe bonds are the shortest, and the Fe–Al bonds have an intermediate length between the lengths of bonds formed by two aluminum atoms and two iron atoms. This relationship seems to be real due to the atomic radii of Al (143 pm) and Fe (126 pm). As for the plane bond angles formed by metal–metal bonds, they are, as a rule, relatively small and almost always less than 90°; a similar situation occurs for planar non-bonded angles, as well as for dihedral (torsion) angles.

As can be seen from the data in Table 1, Table 3, Table 5, Table 7, and Table 9, the most energetically stable metal clusters Al_5_Fe (2-I) and Al_4_Fe_2_ (1-I) are low-spin, while the most energetically stable metal clusters Al_3_Fe_3_ (6-I) and Al_2_Fe_4_ (5-I) are high-spin; the AlFe_5_ (4-I) metal cluster occupies an intermediate position. This fact is quite understandable because the number of unpaired electrons in a neutral Al atom (the electronic configuration of the ground state 3*s*^2^3*p*^1^) is significantly lower than the number of unpaired electrons in a neutral Fe (4*s*^2^3*d*^6^) atom and with an increase in the number of Fe atoms (and, accordingly, a decrease number of Al atoms) in the molecular structure of the Al_n_Fe_m_ metal cluster (n + m = 6), logically, there should be an increase in the total number of unpaired electrons and a higher probability of the realization of the high-spin ground state compared to the low-spin one. Such a prediction, however, is not fully justified when going from Al_2_Fe_4_ to AlFe_5_, because, contrary to our expectations, the ground state of the most stable AlFe_5_ metal cluster, which is AlFe_5_ (4-I), was not in a high-spin state with *M_S_* = 6, but a state with *M_S_* = 4. The reason for such a metamorphosis is apparently related to the fact that in the smallest homohexa-nuclear metal cluster Fe_6_, as the calculation shows, the ground state is the spin singlet (*M_S_* = 1), and the spin multiplicity of the ground state for AlFe_5_ should thus be intermediate, between those of Al_2_Fe_4_ and Fe_6_, which is confirmed by the calculation results. According to NBO analysis data, the charges on the Al and Fe atoms that make up the investigated metal clusters, on the whole, are relatively small and do not exceed 1.00 in absolute value. As should be expected, for the metal clusters presented in Table 11, the charges on the Al atoms are positive, and those on the Fe atoms are negative. This is also quite understandable since the electronegativity of Al on the Pauling scale (1.5) is lower than the electronegativity of Fe (1.8). An interesting exception is the AlFe_5_ (4-I) cluster, in which one of the Fe atoms, namely Fe2, has a positive charge (Table 12). As a rule, the magnitudes of the charges on different atoms of the same element are different, which is also quite obvious because of the asymmetry of most structural isomers of these metal clusters.

According to our calculation, the values of the Gibbs free energy of formation Δ_f_*G*^0^ (298 K), even for the most stable Al_n_Fe_m_ metal clusters (n + m = 6), are positive (Table 11), which means that none of them can be obtained by direct interactions between metallic iron and metallic aluminum. However, the situation changes radically if gaseous aluminum and iron are taken as starting materials, i.e., the following reactions (1)–(5) are used:

5Al(gas) + Fe(gas) → Al_5_Fe(gas) (1)

4Al(gas) + 2Fe(gas) → Al_4_Fe_2_(gas) (2)

3Al(gas) + 3Fe(gas) → Al_3_Fe_3_(gas) (3)

2Al(gas) + 4Fe(gas) → Al_2_Fe_4_(gas) (4)

Al(gas) + 5Fe(gas) → AlFe_5_(gas) (5)

each of which, according to the calculations, can be thermodynamically resolved and belongs to the number of chemical processes occurring with the so-called enthalpy factor (Table 13).

As can be seen from these values, for each of the reactions (1)–(5) in the gas phase, the values of both of these parameters are negative for any of the considered metal clusters; hence, all these reactions are thermodynamically allowed at relatively low temperatures and forbidden at high ones. It is noteworthy that all of these reactions are exothermic. Moreover, the thermal effect of each of them is quite significant. In the simplest version using the Gibbs–Helmholtz Equation (6) for the isobaric process,

Δ_r_*G*^0^ (*T*) = Δ_r_*H*^0^ (298 K) − *T*Δ_r_*S*^0^ (298 K) (6)

where Δ_r_*H*^0^ (298 K) and Δ_r_*S*^0^ (298 K) are the changes in the enthalpy and entropy as a result of a chemical process in standard conditions, respectively; *T* is the process temperature in K; and Δ_r_*G*^0^ (*T*) is the dependence of the Gibbs free energy on the temperature *T*). It is easy to find the temperature at which one or another of the Reactions (1)–(5) will not take place due to thermodynamic prohibition. Actually, this parameter is the temperature indicating the beginning of thermal destruction of a metal cluster (*T*_td_, K) or (*t*_td_, °C) in the gas phase; the values of this parameter for each of the most energy-stable metal clusters, namely, Al_5_Fe (2-I), Al_4_Fe_2_ (1-I), Al_3_Fe_3_ (6-I), Al_2_Fe_4_ (5-I), and AlFe_5_ (4-I), are displayed in Table 14.

As can be seen from the given table, this temperature is very high and for each of the most stable Al_n_Fe_m_ metal clusters (n + m = 6), it exceeds 2000 °C, so at least in this aggregation state, all of them are very resistant to thermal effects. These data also demonstrate that the most stable in this respect is Al_3_Fe_3_ (6-I), and the least stable is Al_5_Fe (2-I).

It seems appropriate to compare the results for the Al_3_Fe_3_ and Al_2_Fe_4_ metal clusters presented in this article and the results for the same metal clusters obtained using a simpler version of the DFT method, namely, DFT OPBE/TZVP (they are presented in a recently published review [33], as well as in earlier original publications [30,31,32]). According to these data, the Al_3_Fe_3_ metal cluster exists in 20 different structural isomers, whereas the Al_2_Fe_4_ metal cluster only exists in nine different structural isomers. These numbers of structural isomers are in sharp contrast to the analogous values for the data of metal clusters obtained using the DFT OPBE/QZVP method (40 and 53, respectively). It should be noted that for the Al_4_Fe_2_ metal cluster, DFT OPBE/TZVP gives practically the same number of possible structural isomers (38) as for the DFT OPBE/QZVP method (39); a similar coincidence can be observed for the Al_5_Fe metal cluster (any data on these two metal clusters, however, were not published by the authors of this article or any other researchers). Therefore, the difference in the number of possible structural isomers obtained by these two methods increases with an increase in the number of 3D-element atoms (in our case, Fe) in the structural unit of the Al_n_Fe_m_ metal cluster. This difference is quite understandable because the DFT OPBE/QZVP method better takes into account the specifics of the electronic structure and wave functions of 3D-element atoms than DFT OPBE/TZVP, and the larger the fraction of Fe atoms in the structure, the more significant the difference in the data should be. It should be noted that, in this regard, the molecular structures of the most stable metal clusters Al_3_Fe_3_ and Al_2_Fe_4_, as well as the spin multiplicities of their ground states, obtained by the DFT OPBE/TZVP method, are similar to the analogous data obtained by the DFT OPBE/QZVP method and presented in this article.

## 4. Calculation Method

The quantum-chemical calculations of Al_n_Fe_m_ metal clusters were carried out using the density functional method (DFT) combining the standard extended split-valence QZVP basis [34,35] and the OPBE functional [36,37]. The data of works [34,35,36,37,38,39,40,41] gave us reason to assert that the given method allows the most accurate estimation of the ratio between energies of the high-spin state and low-spin state to be obtained and, at the same time, rather reliably predicts the key geometric parameters of molecular structures for various compounds of 3*p*- and 3*d*-elements. To build quantum chemical models of the molecular structures of the metal clusters under examination, GAUSSIAN09 software was used [42]. As in [16,17,18,19,20,21,22], the accordance of the found stationary points to the energy minima was confirmed by the calculation of the second derivatives with respect to the atomic coordinates. Additionally, all equilibrium structures corresponding to the minima at the potential energy surface only revealed real positive frequency values. Parameters of the molecular structures for spin multiplicities (*M_S_*) higher than 1 were determined using the so-called unrestricted method (UOPBE), and those for *M_S_* = 1, using the so-called restricted method (*ROPBE*). Along with this, the unrestricted method in conjunction with the GUESS = Mix option was used for the cases when *M_S_* was equal to 1. Moreover, NBO analysis of these metal clusters (Natural Population, Natural Electron Configurations, and Natural Atomic Orbital Occupancies) was carried out according to the procedure described in the works [43,44]. NBO 3.0 version built-in GAUSSIAN09 was used. The energetically most favorable structure has always been checked with the STABLE = OPT procedure; in all cases, the wave function corresponding to it was stable. The standard thermodynamic parameters of the formation of metal clusters under study, and namely Δ_f_*H*^0^ (298 K), *S_f_*^0^ (298 K), and Δ_f_*G*^0^ (298 K) for the metal clusters under examination, were calculated using the method described in [45].

## 5. Conclusions

As can be seen from the calculated data, each of the Al_n_Fe_m_ hexa-nuclear metal clusters (n + m = 6) under examination has a rather significant number of structural isomers, which differ very significantly in terms of their structural and geometric parameters and relative total energies; the total number of such structural isomers increases from Al_5_Fe to Al_2_Fe_4_, while from Al_2_Fe_4_ to AlFe_5_, it decreases. It is also noteworthy that in the case of the so-called “inverted” metal clusters [(Al_5_Fe, AlFe_5_) and (Al_4_Fe_2_, Al_2_Fe_4_)], the number of structural isomers in AlFe_5_ and Al_2_Fe_4_ is noticeably higher than the number of structural isomers in Al_5_Fe and Al_4_Fe_2_, respectively (although, given the uniformity of their composition in each of these two pairs, the same number of these same isomers would be expected). As a rule, structural isomers of the analyzed metal clusters are either completely devoid of symmetry elements, or only have one plane of symmetry; at the same time, it is remarkable that, in most of these compounds, Al–Al chemical bonds are absent. For the spin multiplicity of the most stable metal clusters’ ground state in the series Al_5_Fe–Al_4_Fe_2_–Al_3_Fe_3_–Al_2_Fe_4_–AlFe_5_, the tendency to transition from the low-spin ground state in Al_5_Fe and Al_4_Fe_2_ to the high-spin number of unpaired atoms in Al_3_Fe_3_ и Al_2_Fe_4_ is quite clearly expressed. Taking into account the presence of a greater number of unpaired electrons in the Fe atom in comparison with that in the Al atom, this seems to be quite natural. The most stable Al_n_Fe_m_ metal clusters (n + m = 6) are characterized by a very high thermal stability, although none of them can be directly obtained from metallic iron and aluminum (since, for any of them, the values of the standard Gibbs energies of formation Δ_f_*G*^0^ (298 K) > 0).

In conclusion, the (AlFe) metal clusters considered in the given article could be primarily used in the creation of new composite materials and alloys based on polymetallic nanoparticles in the future; it is quite possible that such alloys may have extraordinary magneto-chemical and physical-mechanical characteristics. Other possible areas of their application may be the doping of traditional alloys based on both non-ferrous and ferrous metals, metal complex catalysis, the creation of specific electrochemical systems, and semiconductor technology. It is also highly probable that they can be used as potential so-called quantum dots, the possibilities of technologies with the use of which are still far from exhausted.

## Figures and Tables

**Table 1:** Relative energies and spin multiplicities of the ground states of various structural isomers of metal clusters with an Al_5_Fe composition.

Structure Designation	Relative Energy, kJ/mol	Structure Designation	Relative Energy, kJ/mol
Metal clusters with *M_S_* = 2		Metal clusters with *M_S_* = 4	
Al_5_Fe (2-I)	0.0	Al_5_Fe (4-I)	12.0
Al_5_Fe (2-II)	4.4	Al_5_Fe (4-II)	14.0
Al_5_Fe (2-III)	23.3	Al_5_Fe (4-III)	16.0
Al_5_Fe (2-IV)	28.9	Al_5_Fe (4-IV)	21.1
Al_5_Fe (2-V)	32.3	Al_5_Fe (4-V)	23.8
Al_5_Fe (2-VI)	54.3	Al_5_Fe (4-VI)	61.9
Al_5_Fe (2-VII)	62.7	Al_5_Fe (4-VII)	78.7
Al_5_Fe (2-VIII)	104.4	Al_5_Fe (4-VIII)	105.2
Al_5_Fe (2-IX)	125.1	Al_5_Fe (4-IX)	105.9
Al_5_Fe (2-X)	126.3		

**Table 2:** Key structural parameters of the most stable Al_5_Fe metal clusters.

Metal Cluster	Al_5_Fe (2-I)	Al_5_Fe (2-II)	Al_5_Fe (4-I)	Al_5_Fe (4-II)	Al_5_Fe (4-III)
Distances between metal atoms, *pm*					
Al1Al2	273.2	284.8	266.7	260.4	274.8
Al1Al3	260.1	271.0	263.0	295.1	271.0
Al2Al3	443.9	424.3	421.2	437.2	377.5
Al1Fe1	242.4	238.0	240.0	240.5	242.8
Al1Al4	279.2	251.4	262.0	277.2	272.5
Al1Al5	279.9	303.3	266.5	262.9	377.7
Al2Fe1	395.2	239.1	362.2	262.3	242.8
Al2Al4	256.2	284.7	254.7	263.5	272.4
Al2Al5	273.2	258.1	259.9	452.5	271.2
Al3Fe1	246.8	442.1	241.2	247.4	429.5
Al3Al4	431.7	270.8	432.6	260.0	267.7
Al3Al5	260.7	259.3	254.8	255.6	251.5
Fe1Al4	244.3	238.0	241.2	253.1	237.5
Fe1Al5	242.5	415.0	362.1	251.6	429.5
Al4Al5	279.1	303.2	421.0	422.2	267.5
Plane angles between metal atoms, *deg.*					
Al4Al1Al5	59.9	65.5	105.3	102.8	45.1
Fe1Al1Al4	55.3	58.1	57.1	58.0	54.5
Fe1Al2Al3	33.6	78.1	34.8	30.0	84.7
Al4Al2Al3	70.4	39.0	75.1	33.1	45.2
Al1Al2Al4	63.6	52.4	60.5	63.9	59.7
Al4Al1Al2	55.3	63.8	57.5	58.6	59.7
Al1Al2Al3	32.7	39.0	37.0	41.0	45.8
Al2Al3Fe1	62.3	32.0	59.1	32.0	34.3
Al3Fe1Al2	84.1	69.9	86.1	118.1	61.1
Al5Al1Al2	59.2	51.9	58.3	119.7	45.8
Fe1Al1Al5	54.8	99.4	91.1	59.8	84.7
Fe1Al1Al2	99.9	53.5	91.1	63.0	55.5
Al1Fe1Al2	42.9	73.3	47.4	62.2	68.9
Al3Al4Al5	35.3	53.3	34.7	34.7	56.0

**Table 3:** Relative energies and spin multiplicities of the ground states of various structural isomers of metal clusters with an Al_4_Fe_2_ composition.

Structure Designation	Relative Energy, kJ/mol	Structure Designation	Relative Energy, kJ/mol
Metal clusters with *M_S_* = 1		Al_4_Fe_2_ (3-IV)	57.2
Al_4_Fe_2_ (1-I)	0.0	Al_4_Fe_2_ (3-V)	58.5
Al_4_Fe_2_ (1-II)	93.4	Al_4_Fe_2_ (3-VI)	59.6
Al_4_Fe_2_ (1-III)	95.0	Al_4_Fe_2_ (3-VII)	62.4
Al_4_Fe_2_ (1-IV)	101.9	Al_4_Fe_2_ (3-VIII)	65.0
Al_4_Fe_2_ (1-V)	103.3	Al_4_Fe_2_ (3-IX)	82.2
Al_4_Fe_2_ (1-VI)	132.5	Al_4_Fe_2_ (3-X)	111.5
Al_4_Fe_2_ (1-VII)	135.9	Al_4_Fe_2_ (3-XI)	218.4
Al_4_Fe_2_ (1-VIII)	139.5	Al_4_Fe_2_ (3-XII)	229.7
Al_4_Fe_2_ (1-IX)	147.7	Al_4_Fe_2_ (3-XIII)	246.8
Al_4_Fe_2_ (1-X)	155.3	Metal clusters with *M_S_* = 5	
Al_4_Fe_2_ (1-XI)	169.2	Al_4_Fe_2_ (5-I)	22.1
Al_4_Fe_2_ (1-XII)	169.3	Al_4_Fe_2_ (5-II)	22.5
Al_4_Fe_2_ (1-XIII)	183.4	Al_4_Fe_2_ (5-III)	30.8
Al_4_Fe_2_ (1-XIV)	205.7	Al_4_Fe_2_ (5-IV)	32.2
Al_4_Fe_2_ (1-XV)	211.8	Al_4_Fe_2_ (5-V)	32.8
Al_4_Fe_2_ (1-XVI)	254.3	Al_4_Fe_2_ (5-VI)	34.3
Metal clusters with *M_S_* = 3		Al_4_Fe_2_ (5-VII)	51.0
Al_4_Fe_2_ (3-I)	24.0	Al_4_Fe_2_ (5-VIII)	60.6
Al_4_Fe_2_ (3-II)	25.0	Al_4_Fe_2_ (5-IX)	64.6
Al_4_Fe_2_ (3-III)	40.0	Al_4_Fe_2_ (5-X)	69.9

**Table 4:** Key structural parameters of the most stable Al_4_Fe_2_ metal clusters.

Metal Cluster	Al_4_Fe_2_ (1-I)	Al_4_Fe_2_ (5-I)	Al_4_Fe_2_ (5-II)	Al_4_Fe_2_ (3-I)	Al_4_Fe_2_ (3-II)
Distances between metal atoms, *pm*					
Al1Al2	256.6	265.0	288.8	432.8	261.9
Al1Al3	362.9	277.3	335.9	277.8	275.6
Al2Al3	256.6	277.2	443.2	267.9	275.8
Al1Fe1	248.9	244.1	246.8	241.2	246.5
Al1Fe2	248.9	244.1	246.8	252.7	246.5
Al1Al4	256.6	277.3	443.2	429.9	275.6
Al2Fe1	248.9	244.1	246.8	250.4	246.5
Al2Fe2	248.9	244.1	246.8	244.6	246.5
Al2Al4	362.9	277.3	336.3	266.3	275.6
Al3Fe1	248.9	245.3	246.8	238.0	238.2
Al3Fe2	248.9	403.9	246.8	249.9	399.8
Al3Al4	256.6	483.5	288.8	264.5	483.8
Fe1Fe2	340.8	212.9	217.5	214.8	213.1
Fe1Al4	248.9	403.9	246.8	393.5	399.8
Fe2Al4	248.9	245.3	246.8	244.4	238.2
Plane angles between metal atoms, deg.					
Fe2Al1Al4	59.0	55.7	26.1	29.6	53.9
Fe1Al1Fe2	86.4	51.7	52.3	51.5	51.2
Fe1Al2Al3	59.0	55.7	26.1	54.5	53.9
Fe2Al2Al3	59.0	101.4	26.1	58.2	99.8
Al1Al2Fe2	59.0	57.1	54.2	30.0	57.9
Fe2Al1Al2	59.0	57.1	54.2	29.0	57.9
Al1Al2Al3	90.0	61.5	49.3	38.3	61.6
Al2Al3Fe1	59.0	55.3	26.1	59.0	56.8
Al3Fe1Al2	62.1	69.0	127.7	66.5	69.3
Al4Al1Al2	90.0	61.5	49.4	36.0	61.6
Fe1Al1Al4	59.0	101.4	26.1	64.8	99.8
Fe1Al1Al2	59.0	57.1	54.2	28.9	57.9
Al1Fe1Al2	62.1	65.8	71.6	125.3	64.2
Al3Fe2Al4	62.1	93.0	71.6	119.8	95.3

**Table 5:** Relative energies and spin multiplicities of the ground states of various structural isomers of metal clusters of Al_3_Fe_3_.

Structure Designation	Relative Energy, kJ/mol	Structure Designation	Relative Energy, kJ/mol
Metal clusters with *M_S_* = 2		Al_3_Fe_3_ (4-IV)	102.4
Al_3_Fe_3_ (2-I)	51.6	Al_3_Fe_3_ (4-V)	103.4
Al_3_Fe_3_ (2-II)	72.4	Al_3_Fe_3_ (4-VI)	106.6
Al_3_Fe_3_ (2-III)	82.2	Al_3_Fe_3_ (4-VII)	114.5
Al_3_Fe_3_ (2-IV)	89.2	Al_3_Fe_3_ (4-VIII)	119.1
Al_3_Fe_3_ (2-V)	94.6	Al_3_Fe_3_ (4-IX)	122.4
Al_3_Fe_3_ (2-VI)	94.8	Al_3_Fe_3_ (4-X)	124.7
Al_3_Fe_3_ (2-VII)	102.9	Al_3_Fe_3_ (4-XI)	129.6
Al_3_Fe_3_ (2-VIII)	108.4	Al_3_Fe_3_ (4-XII)	147.2
Al_3_Fe_3_ (2-IX)	114.8	Al_3_Fe_3_ (4-XIII)	162.5
Al_3_Fe_3_ (2-X)	122.3	Metal clusters with *M_S_* = 6	
Al_3_Fe_3_ (2-XI)	125.6	Al_3_Fe_3_ (6-I)	0.0
Al_3_Fe_3_ (2-XII)	132.2	Al_3_Fe_3_ (6-II)	3.5
Al_3_Fe_3_ (2-XIII)	134.6	Al_3_Fe_3_ (6-III)	32.7
Al_3_Fe_3_ (2-XIV)	136.5	Al_3_Fe_3_ (6-IV)	57.4
Al_3_Fe_3_ (2-XV)	137.1	Al_3_Fe_3_ (6-V)	61.0
Al_3_Fe_3_ (2-XVI)	154.0	Al_3_Fe_3_ (6-VI)	65.9
Al_3_Fe_3_ (2-XVII)	174.9	Al_3_Fe_3_ (6-VII)	67.6
Metal clusters with *M_S_* = 4		Al_3_Fe_3_ (6-VIII)	79.2
Al_3_Fe_3_ (4-I)	49.1	Al_3_Fe_3_ (6-IX)	86.5
Al_3_Fe_3_ (4-II)	81.2	Al_3_Fe_3_ (6-X)	99.6
Al_3_Fe_3_ (4-III)	89.0		

**Table 6:** Key structural parameters of the most stable Al_3_Fe_3_ metal clusters.

Metal Cluster	Al_3_Fe_3_ (6-I)	Al_3_Fe_3_ (6-II)	Al_3_Fe_3_ (6-III)	Al_3_Fe_3_ (4-I)	Al_3_Fe_3_ (2-I)
Distances between metal atoms, *pm*					
Al1Al2	305.1	270.6	262.3	259.8	268.8
Al1Al3	305.8	270.7	262.3	258.4	268.8
Al2Al3	398.5	368.5	384.3	372.3	374.3
Al1Fe1	236.0	241.8	271.4	245.7	241.0
Al1Fe2	369.4	354.8	245.5	346.1	338.0
Al1Fe3	236.0	241.8	245.5	243.7	243.8
Al2Fe1	244.0	239.7	242.8	251.0	250.8
Al2Fe2	242.3	241.8	389.6	252.5	236.8
Al2Fe3	244.0	239.7	249.2	242.8	248.2
Al3Fe1	244.0	239.7	242.8	248.5	250.8
Al3Fe2	242.3	241.8	249.2	246.8	236.8
Al3Fe3	244.0	239.7	389.6	253.0	248.2
Fe1Fe2	222.3	226.8	219.1	221.5	235.4
Fe1Fe3	270.9	305.7	219.1	330.4	328.7
Fe2Fe3	222.3	220.8	253.4	246.3	222.9
Plane angles between metal atoms, *deg.*					
Fe2Al1Fe3	35.0	39.2	56.7	45.4	41.2
Fe1Al1Fe2	35.0	39.2	49.8	39.6	44.2
Fe1Al2Al3	35.2	39.8	37.7	41.6	41.7
Fe2Al2Al3	34.7	40.4	37.6	41.2	37.8
Al1Al2Fe2	84.1	87.4	38.3	85.0	83.6
Fe2Al1Al2	40.7	42.9	100.2	46.6	44.1
Al1Al2Al3	49.4	47.1	42.9	43.9	45.9
Al2Al3Fe1	35.2	39.8	37.7	42.1	41.7
Al3Fe1Al2	109.5	100.5	105.6	96.4	96.5
Fe3Al1Al2	51.7	55.4	58.7	57.6	57.7
Fe1Al1Fe3	70.1	78.4	49.8	84.9	85.4
Fe1Al1Al2	51.7	55.4	54.1	59.5	58.6
Al1Fe1Al2	78.9	68.4	61.0	63.1	66.2
Al3Fe2Fe3	63.2	61.4	107.6	61.8	65.3

**Table 7:** Relative energies and spin multiplicities of the ground states of various structural isomers of metal clusters of Al_2_Fe_4_.

Structure Designation	Relative Energy, kJ/mol	Structure Designation	Relative Energy, kJ/mol	Structure Designation	Relative Energy, kJ/mol
Metal clusters with *M_S_* = 1		Al_2_Fe_4_ (1-XIX)	359.2	Al_2_Fe_4_ (3-IX)	64.3
Al_2_Fe_4_ (1-I)	15.3	Al_2_Fe_4_ (1-XX)	378.3	Al_2_Fe_4_ (3-X)	77.3
Al_2_Fe_4_ (1-II)	15.9	Al_2_Fe_4_ (1-XXI)	378.7	Al_2_Fe_4_ (3-XI)	86.8
Al_2_Fe_4_ (1-III)	16.1	Al_2_Fe_4_ (1-XXII)	394.4	Al_2_Fe_4_ (3-XII)	89.4
Al_2_Fe_4_ (1-IV)	17.2	Al_2_Fe_4_ (1-XXIII)	394.6	Al_2_Fe_4_ (3-XIII)	91.2
Al_2_Fe_4_ (1-V)	30.1	Al_2_Fe_4_ (1-XXIV)	402.1	Al_2_Fe_4_ (3-XIV)	121.1
Al_2_Fe_4_ (1-VI)	41.9	Al_2_Fe_4_ (1-XXV)	403.0	Al_2_Fe_4_ (3-XV)	201.7
Al_2_Fe_4_ (1-VII)	53.8	Al_2_Fe_4_ (1-XXVI)	403.3	Metal clusters with *M_S_* = 5	
Al_2_Fe_4_ (1-VIII)	83.1	Al_2_Fe_4_ (1-XXVII)	409.8	Al_2_Fe_4_ (5-I)	0.0
Al_2_Fe_4_ (1-IX)	129.3	Al_2_Fe_4_ (1-XXVIII)	426.0	Al_2_Fe_4_ (5-II)	16.8
Al_2_Fe_4_ (1-X)	142.6	Metal clusters with *M_S_* = 3		Al_2_Fe_4_ (5-III)	24.4
Al_2_Fe_4_ (1-XI)	187.3	Al_2_Fe_4_ (3-I)	15.0	Al_2_Fe_4_ (5-IV)	29.0
Al_2_Fe_4_ (1-XII)	231.1	Al_2_Fe_4_ (3-II)	15.7	Al_2_Fe_4_ (5-V)	41.2
Al_2_Fe_4_ (1-XIII)	241.9	Al_2_Fe_4_ (3-III)	26.6	Al_2_Fe_4_ (5-VI)	45.2
Al_2_Fe_4_ (1-XIV)	259.0	Al_2_Fe_4_ (3-IV)	27.6	Al_2_Fe_4_ (5-VII)	119.8
Al_2_Fe_4_ (1-XV)	259.4	Al_2_Fe_4_ (3-V)	37.6	Al_2_Fe_4_ (5-VIII)	131.1
Al_2_Fe_4_ (1-XVI)	330.8	Al_2_Fe_4_ (3-VI)	44.2	Al_2_Fe_4_ (5-IX)	204.2
Al_2_Fe_4_ (1-XVII)	350.3	Al_2_Fe_4_ (3-VII)	56.8	Al_2_Fe_4_ (5-X)	212.1
Al_2_Fe_4_ (1-XVIII)	352.4	Al_2_Fe_4_ (3-VIII)	57.5		

**Table 8:** Key structural parameters of the most stable Al_2_Fe_4_ metal clusters.

Metal Cluster	Al_2_Fe_4_ (5-I)	Al_2_Fe_4_ (3-I)	Al_2_Fe_4_ (1-I)	Al_2_Fe_4_ (3-II)	Al_2_Fe_4_ (1-II)
Distances between metal atoms, *pm*					
Fe1Fe2	333.1	244.1	217.0	329.9	218.5
Fe1Fe3	246.1	213.4	252.9	241.9	379.5
Fe2Fe3	226.5	326.5	335.6	214.7	254.4
Fe1Al1	249.7	249.2	242.0	246.6	242.9
Fe1Al2	249.7	249.2	350.2	246.6	242.9
Fe1Fe4	246.1	326.5	248.5	234.3	395.9
Fe2Al1	242.9	247.6	247.7	248.7	250.5
Fe2Al2	242.9	247.7	252.6	248.7	250.5
Fe2Fe4	226.5	220.2	253.0	242.1	389.9
Fe3Al1	242.9	249.2	252.7	248.7	246.1
Fe3Al2	348.7	249.2	247.8	248.7	246.1
Fe3Fe4	231.8	244.1	217.0	330.0	216.7
Al1Al2	270.1	374.5	262.6	369.0	277.5
Al1Fe4	348.6	247.7	350.2	246.6	248.5
Al2Fe4	242.8	247.7	242.0	246.6	248.4
Plane angles between metal atoms, *deg.*					
Al2Fe1Fe4	58.6	48.7	43.7	61.6	36.8
Al1Fe1Al2	65.5	97.4	48.4	96.9	69.7
Al1Fe2Fe3	62.2	49.1	48.5	64.4	58.3
Al2Fe2Fe3	95.9	49.1	47.3	64.4	58.3
Fe1Fe2Al2	48.3	60.9	96.1	48.0	61.9
Al2Fe1Fe2	46.6	60.3	45.8	48.5	65.5
Fe1Fe2Fe3	47.6	40.8	48.9	47.1	106.5
Fe2Fe3Al1	62.2	48.7	47.3	64.4	60.0
Fe3Al1Fe2	55.6	82.2	84.2	51.1	61.6
Fe4Fe1Fe2	42.8	42.4	65.4	47.2	72.4
Al1Fe1Fe4	89.4	48.7	91.1	61.6	36.8
Al1Fe1Fe2	46.6	60.3	65.0	48.5	65.5
Fe1Al1Fe2	85.1	58.9	52.6	83.5	52.6
Fe3Al2Fe4	41.5	58.9	52.6	83.5	52.0

**Table 9:** Relative energies and spin multiplicities of the ground states of various structural isomers of metal clusters of AlFe_5_.

Structure Designation	Relative Energy, kJ/mol	Structure Designation	Relative Energy, kJ/mol
Metal clusters with *M_S_* = 2		AlFe_5_ (4-III)	49.1
AlFe_5_ (2-I)	18.7	AlFe_5_ (4-IV)	123.7
AlFe_5_ (2-II)	57.6	AlFe_5_ (4-V)	131.5
AlFe_5_ (2-III)	65.5	AlFe_5_ (4-VI)	132.2
AlFe_5_ (2-IV)	69.7	AlFe_5_ (4-VII)	162.1
AlFe_5_ (2-V)	123.0	AlFe_5_ (4-VIII)	252.0
AlFe_5_ (2-VI)	139.1	Metal clusters with *M_S_* = 6	
AlFe_5_ (2-VII)	177.4	AlFe_5_ (6-I)	33.8
AlFe_5_ (2-VIII)	178.4	AlFe_5_ (6-II)	61.2
AlFe_5_ (2-IX)	254.7	AlFe_5_ (6-III)	69.4
Metal clusters with *M_S_* = 4		AlFe_5_ (6-IV)	105.1
AlFe_5_ (4-I)	0.0	AlFe_5_ (6-V)	161.9
AlFe_5_ (4-II)	36.0	AlFe_5_ (6-VI)	189.8

**Table 10:** Key structural parameters of the most stable AlFe_5_ metal clusters.

Metal Cluster	AlFe_5_ (4-I)	AlFe_5_ (2-I)	AlFe_5_ (6-I)	AlFe_5_ (4-II)	AlFe_5_ (4-III)
Distances between metal atoms, *pm*					
Fe1Fe2	223.6	224.4	233.1	221.4	251.4
Fe1Fe3	252.1	248.2	249.8	382.4	251.5
Fe2Fe3	252.8	329.9	335.1	393.7	342.7
Fe1Al1	246.1	353.0	358.0	219.5	351.8
Fe1Fe4	231.2	248.3	245.0	233.1	210.7
Fe1Fe5	335.8	224.4	223.9	258.7	251.5
Fe2Al1	350.9	243.6	250.5	249.2	243.7
Fe2Fe4	223.5	245.9	238.1	224.2	252.6
Fe2Fe5	252.7	222.1	228.8	388.9	225.1
Fe3Al1	252.2	252.1	249.4	247.4	243.8
Fe3Fe4	335.8	218.0	212.5	255.7	252.7
Fe3Fe5	212.9	245.7	250.8	211.7	225.1
Al1Fe4	246.1	252.1	244.8	273.7	254.4
Al1Fe5	252.1	243.6	251.1	252.1	248.4
Fe4Fe5	252.0	329.9	323.1	254.0	330.0
Plane angles between metal atoms, *deg.*					
Fe4Fe1Fe5	48.6	88.4	87.0	61.9	90.7
Al1Fe1Fe4	62.0	45.6	43.0	69.0	45.8
Al1Fe2Fe3	45.9	49.4	47.8	37.4	45.4
Fe4Fe2Fe3	89.5	41.4	39.1	37.6	47.3
Fe1Fe2Fe4	62.3	63.5	62.7	63.1	49.4
Fe4Fe1Fe2	58.9	62.5	59.7	59.0	65.6
Fe1Fe2Fe3	63.6	48.8	48.2	70.6	47.1
Fe2Fe3Al1	88.0	47.2	48.1	37.7	45.3
Fe3Al1Fe2	46.0	83.4	84.2	104.9	89.3
Fe5Fe1Fe2	48.8	59.3	60.1	108.0	53.2
Al1Fe1Fe5	48.4	43.1	44.0	59.4	44.9
Al1Fe1Fe2	90.6	43.1	44.1	63.6	43.8
Fe1Al1Fe2	39.8	39.0	40.4	52.7	45.6
Fe3Fe4Fe5	39.3	48.1	50.9	49.1	42.9

**Table 11:** Standard thermodynamic parameters of formation for the most energetically stable (heterobi)hexa-nuclear metal clusters of Al_n_Fe_m_ (n + m = 6).

Metal Cluster	Δ_f_*H*^0^ (298 K), kJ/mol	*S_f_*^0^ (298 K), J/mol K	Δ_f_*G*^0^ (298 K), kJ/mol
Al_5_Fe (2-I)	805.0	457.5	718.9
Al_4_Fe_2_ (1-I)	789.0	445.8	706.1
Al_3_Fe_3_ (6-I)	807.5	469.0	717.4
Al_2_Fe_4_ (5-I)	890.1	480.3	796.3
AlFe_5_ (4-I)	927.6	476.1	834.7

**Table 12:** Charge distribution (in units of electron charge) of various Al and Fe atoms for the most stable Al_n_Fe_m_ metal clusters (n + m = 6) according to NBO analysis data.

Al_5_Fe (2-I)					
Al1	Al2	Al3	Al4	Al5	Fe1
+0.0719	+0.1187	+0.2723	+0.2682	+0.0716	−0.8027
**Al_4_Fe_2_ (1-I)**					
Al1	Al2	Al3	Al4	Fe1	Fe2
+0.1861	+0.1861	+0.1861	+0.1861	−0.3722	−0.3722
**Al_3_Fe_3_ (6-I)**					
Al1	Al2	Al3	Fe1	Fe2	Fe3
+0.5766	+0.6292	+0.6312	−0.7980	−0.2411	−0.7979
**Al_2_Fe_4_ (5-I)**					
Al1	Al2	Fe1	Fe2	Fe3	Fe4
+0.3197	+0.3197	−0.1446	−0.2312	−0.1316	−0.1320
**AlFe_5_ (4-I)**					
Al1	Fe1	Fe2	Fe3	Fe4	Fe5
+0.2554	−0.1544	+0.0823	−0.0161	−0.0782	−0.0890

**Table 13:** Calculated values of the parameters Δ_r_*H*^0^ (298 K) and Δ_r_*S*^0^ (298 K) for the Reactions (1)–(5).

Parameter	Al_5_Fe	Al_4_Fe_2_	Al_3_Fe_3_	Al_2_Fe_4_	AlFe_5_
Δ_r_*H*^0^ (298 K), kJ	−1257.1	−1360.5	−1429.4	−1434.2	−1484.1
Δ_r_*S*^0^ (298 K), J/K	−545.1	−572.7	−565.5	−570.1	−590.2

**Table 14:** Temperatures of the beginning of thermal destruction (*T*_td_, K) and (*t*_td_, °C) of the most energetically-stable metal clusters of Al_n_Fe_m_ (n + m = 6).

Metal Cluster	*T*_td_, K	*t*_td_, °C
Al_5_Fe (2-I)	2306.18	2008.02
Al_4_Fe_2_ (1-I)	2375.58	2077.42
Al_3_Fe_3_ (6-I)	2527.67	2229.51
Al_2_Fe_4_ (5-I)	2515.57	2217.41
AlFe_5_ (4-I)	2514.57	2216.41

## Supplementary Materials

These data are available online at https://www.mdpi.com/1996-1944/14/3/597/s1. Figure S1: Molecular Structures of Hexanuclear Al_5_Fe Metal Clusters calculated by DFT OPBE/QZVP method, Figure S2: Molecular Structures of Hexanuclear Al_4_Fe_2_ Metal Clusters calculated by DFT OPBE/QZVP method, Figure S3: Molecular Structures of Hexanuclear Al_3_Fe_3_ Metal Clusters calculated by DFT OPBE/QZVP method, Figure S4: Molecular Structures of Hexanuclear Al_2_Fe_4_ Metal Clusters calculated by DFT OPBE/QZVP method, Figure S5: Molecular Structures of Hexanuclear AlFe_5_ Metal Clusters calculated by DFT OPBE/QZVP method.
