# Supplementary material for: Models of Molecular Structures of Hexa-Nuclear AlnFem Metal Clusters (n + m = 6): DFT Quantum-Chemical Design
Source: Materials (Basel). 2021 Jan 27;14(3):597. doi: 10.3390/ma14030597 (PMC7865671; doi:10.3390/ma14030597)
Supplement: Supplementary file 1 [file materials-14-00597-s001.zip › materials-1049972-supplementary.pdf]

Molecular Structures
of Hexanuclear Al_5Fe Metal Clusters
calculated by DFT OPBE/QZVP method

Al_5Fe clusters having ground state with M_S= 2

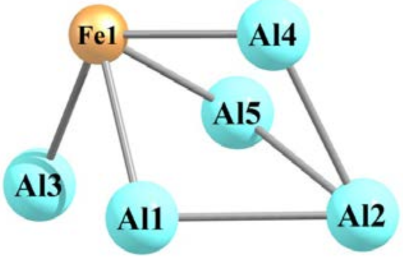

Al_5Fe (2-I)

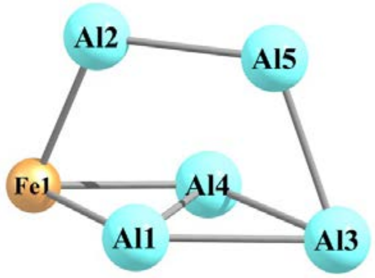

Al_5Fe (2-II)

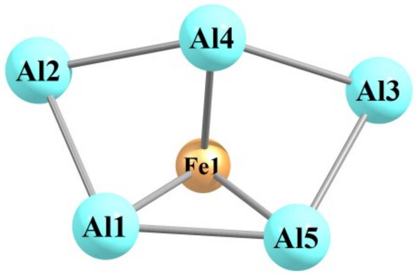

Al_5Fe (2-III)

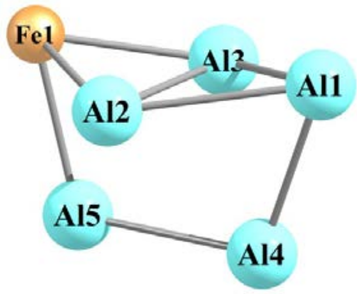

Al_5Fe (2-IV)

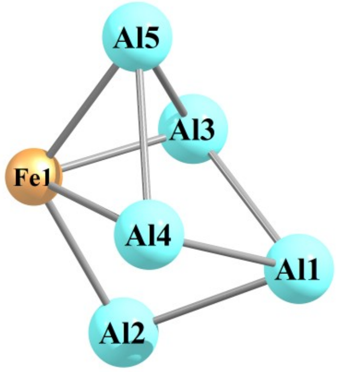

Al_5Fe (2-V)

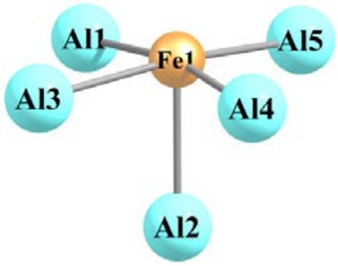

Al_5Fe (2-VI)

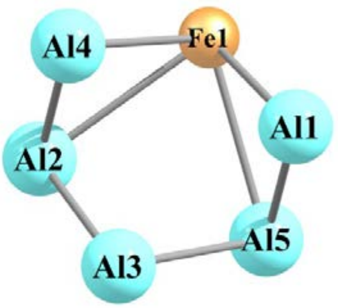

Al_5Fe (2-VII)

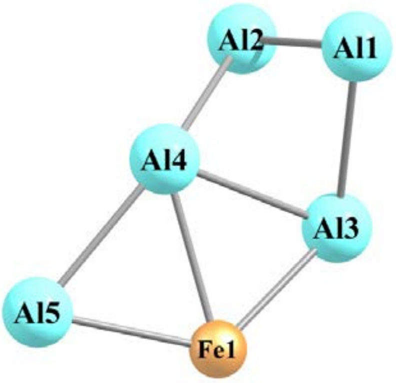

Al_5Fe (2-VIII)

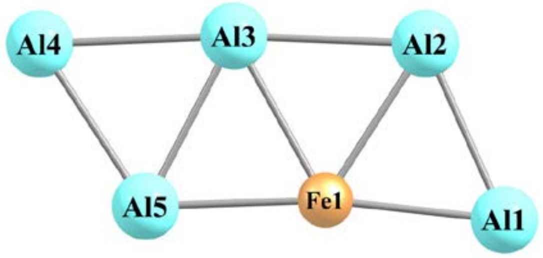

Al_5Fe (2-IX)

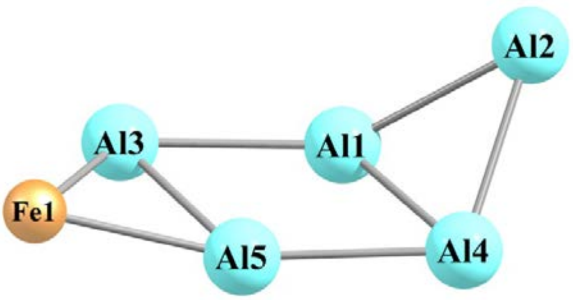

Al_5Fe (2-X)

Al5Fe clusters having ground state with M_S= 4

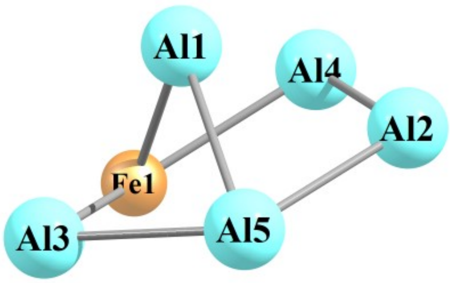

Al_5Fe (4-I)

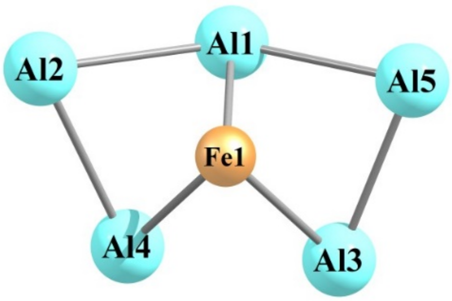

Al_5Fe (4-II)

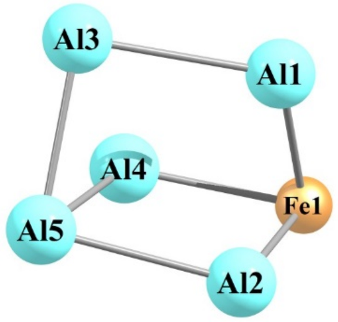

Al_5Fe (4-III)

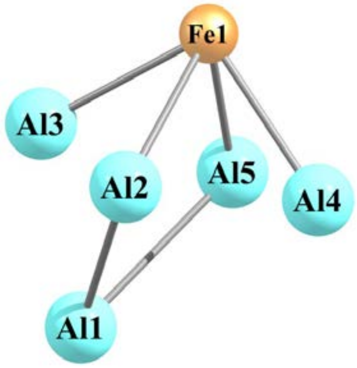

Al_5Fe (4-IV)

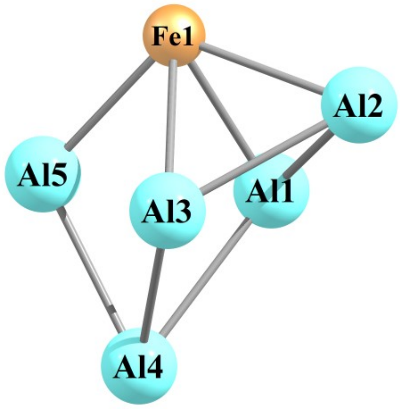

Al_5Fe (4-V)

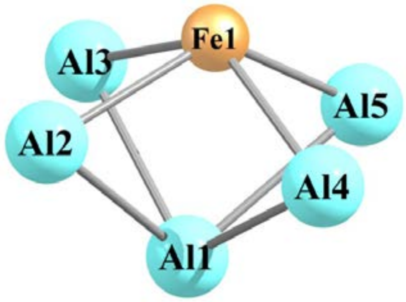

Al_5Fe (4-VI)

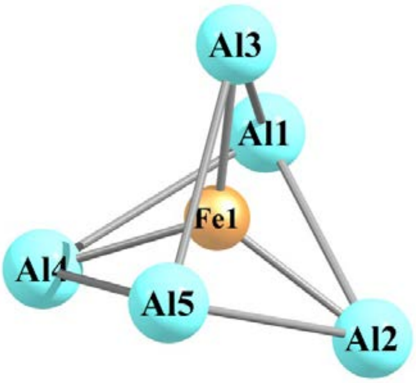

Al_5Fe (4-VII)

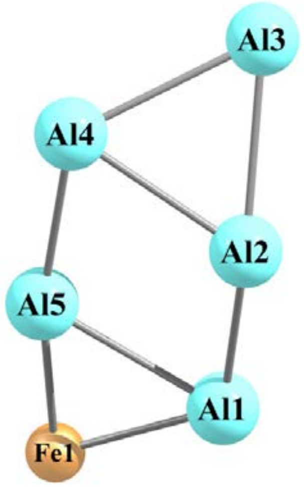

Al_5Fe (4-VIII)

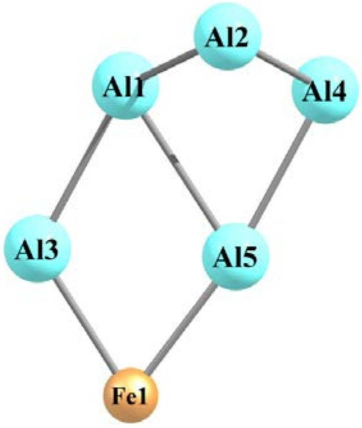

Al_5Fe (4-IX)

Molecular Structures
of Hexanuclear Al_4Fe_2 Metal Clusters
calculated by DFT OPBE/QZVP method

Al_4Fe_2 clusters having ground state with $M_S = 1$

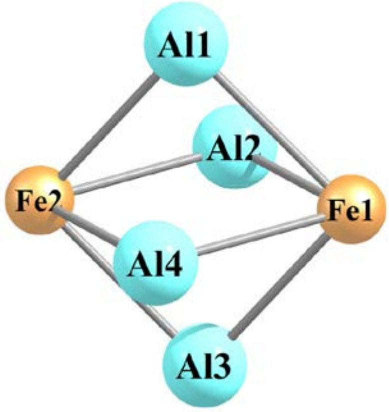

Al_4Fe_2 (1-I)

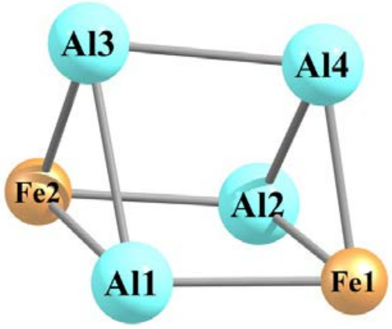

Al_4Fe_2 (1-II)

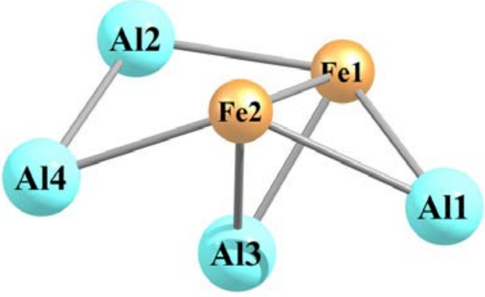

Al_4Fe_2 (1-III)

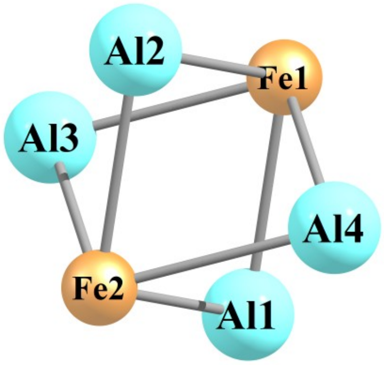

Al_4Fe_2 (1-IV)

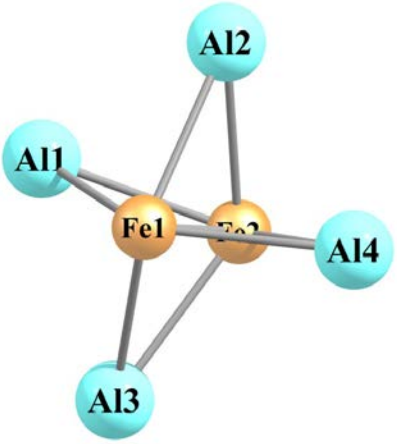

Al_4Fe_2 (1-V)

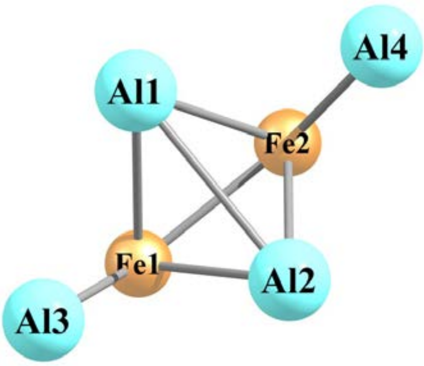

Al_4Fe_2 (1-VI)

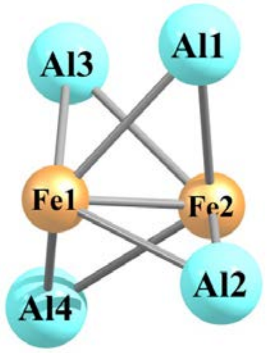

Al_4Fe_2 (1-VII)

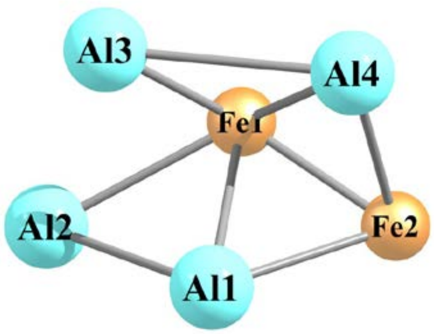

Al_4Fe_2 (1-VIII)

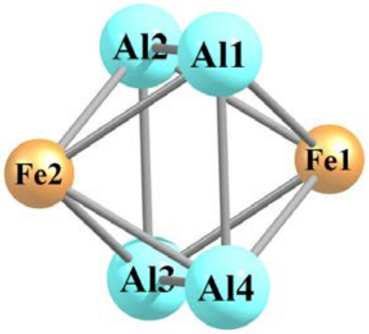

Al_4Fe_2 (1-IX)

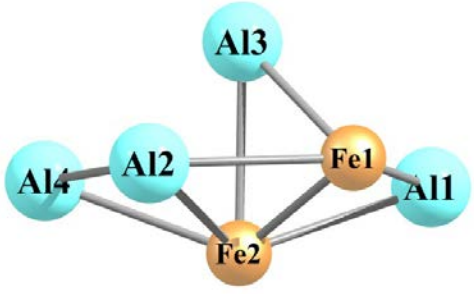

Al_4Fe_2 (1-X)

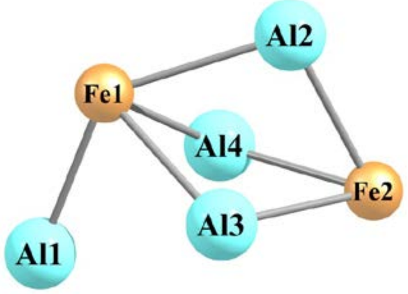

Al_4Fe_2 (1-XI)

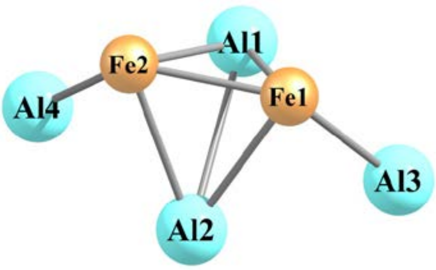

Al_4Fe_2 (1-XII)

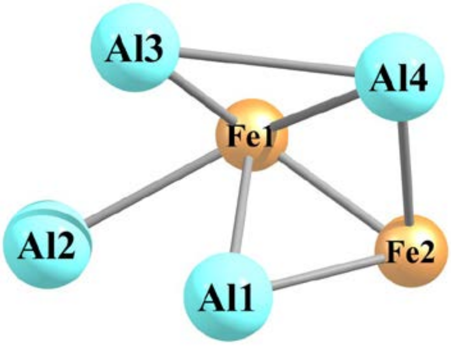

Al_4Fe_2 (1-XIII)

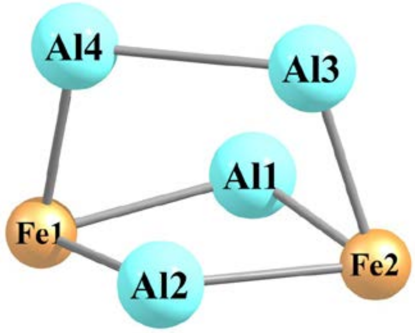

Al_4Fe_2 (1-XIV)

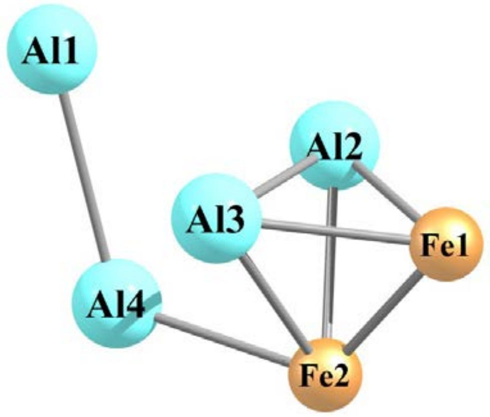

Al_4Fe_2 (1-XV)

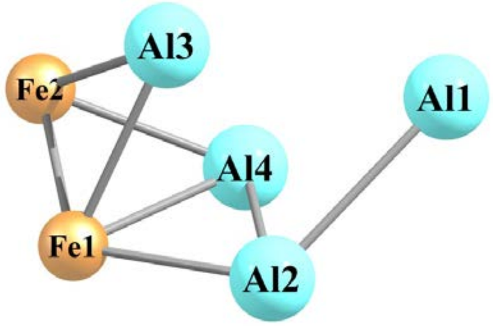

Al_4Fe_2 (1-XVI)

Al_4Fe_2 clusters having ground state with $M_S = 3$

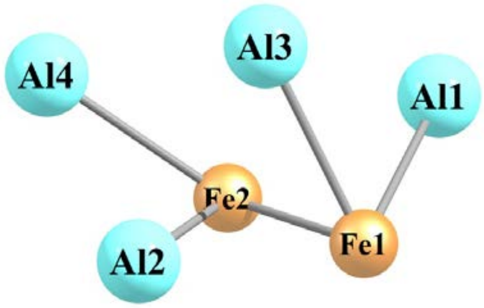

Al_4Fe_2 (3-I)

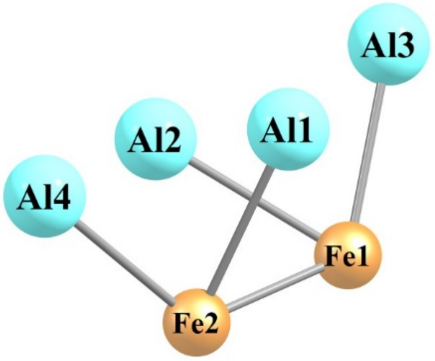

Al_4Fe_2 (3-II)

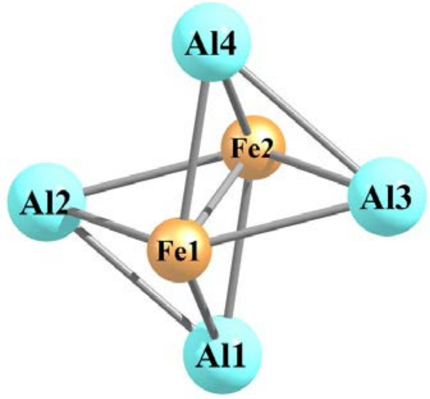

Al_4Fe_2 (3-III)

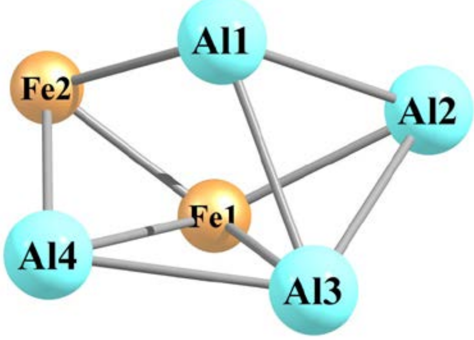

Al_4Fe_2 (3-IV)

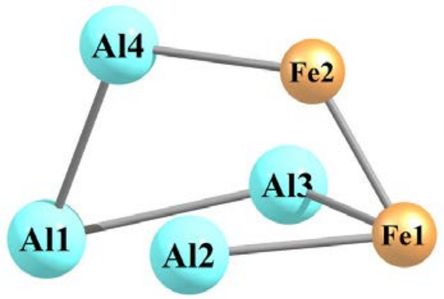

Al_4Fe_2 (3-V)

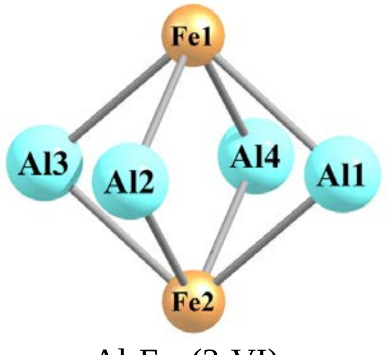

Al_4Fe_2 (3-VI)

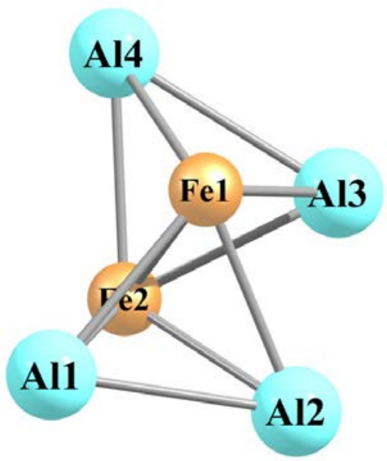

Al_4Fe_2 (3-VII)

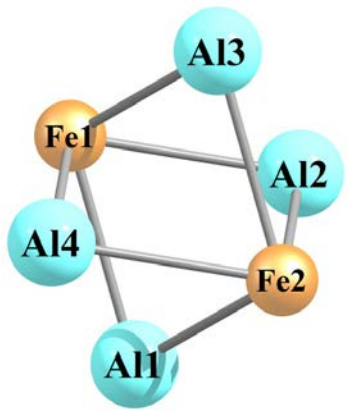

Al_4Fe_2 (3-VIII)

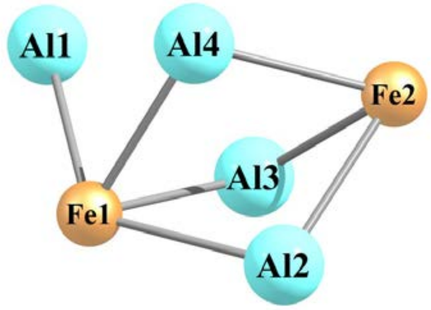

Al_4Fe_2 (3-IX)

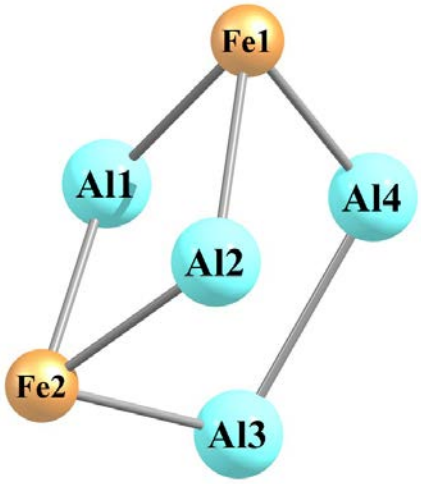

Al_4Fe_2 (3-X)

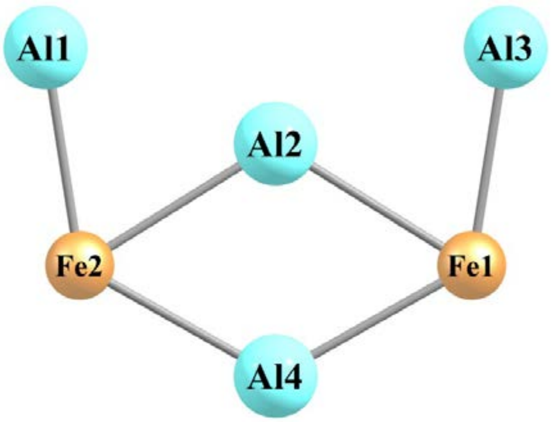

Al_4Fe_2 (3-XI)

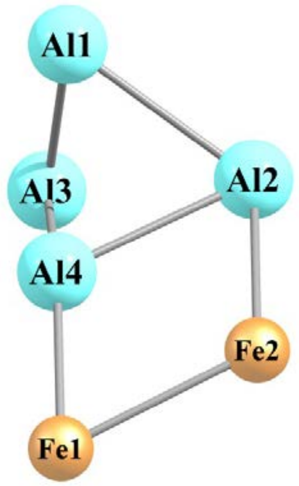

Al_4Fe_2 (3-XII)

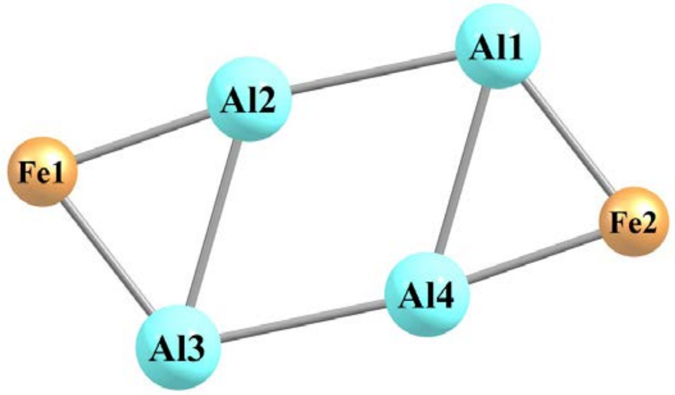

Al_4Fe_2 (3-XIII)

Al_4Fe_2 clusters having ground state with M_S= 5

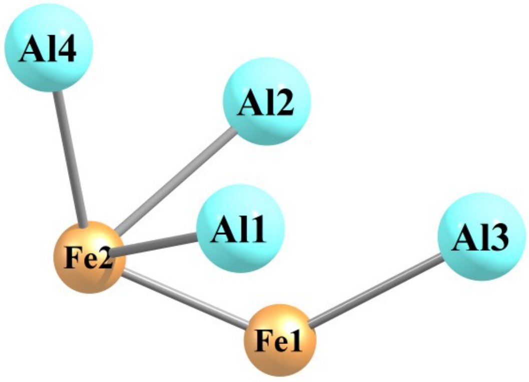

Al_4Fe_2 (5-I)

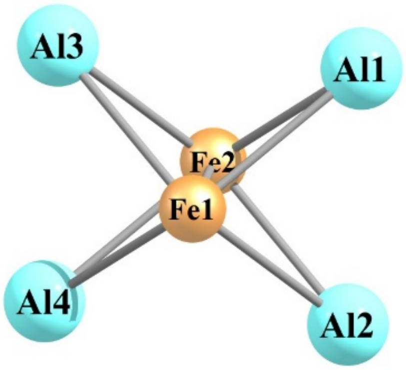

Al_4Fe_2 (5-II)

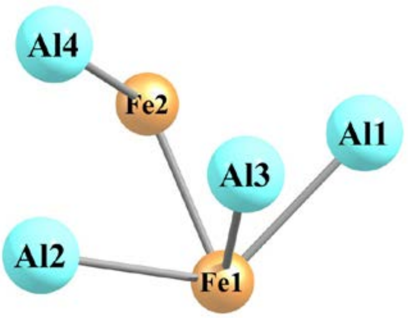

Al_4Fe_2 (5-III)

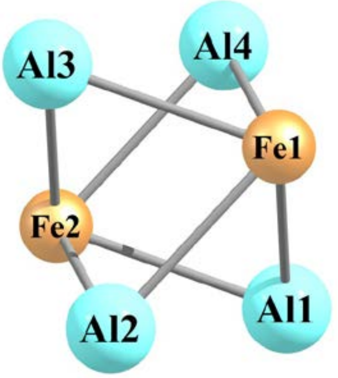

Al_4Fe_2 (5-IV)

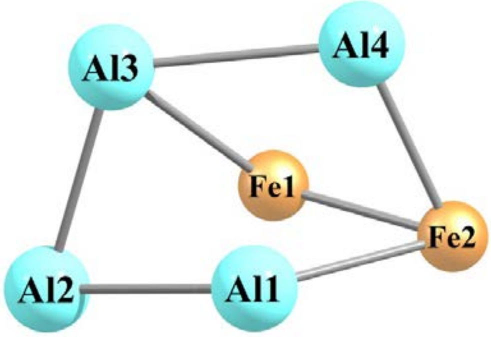

Al_4Fe_2 (5-V)

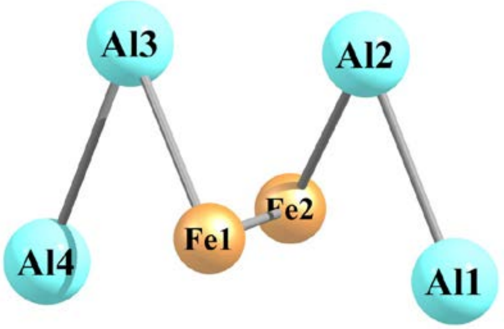

Al_4Fe_2 (5-VI)

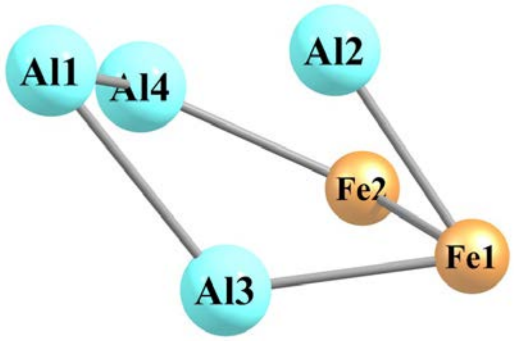

Al_4Fe_2 (5-VII)

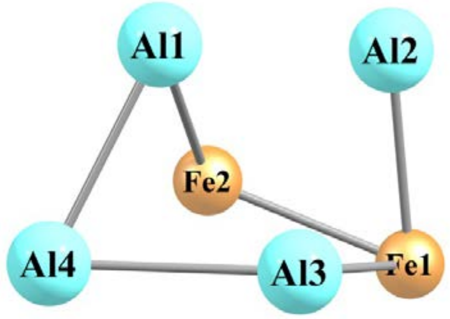

Al_4Fe_2 (5-VIII)

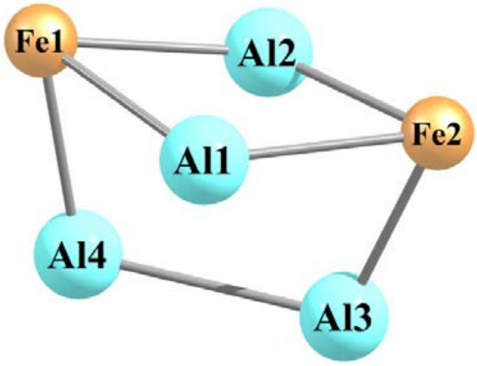

Al_4Fe_2 (5-IX)

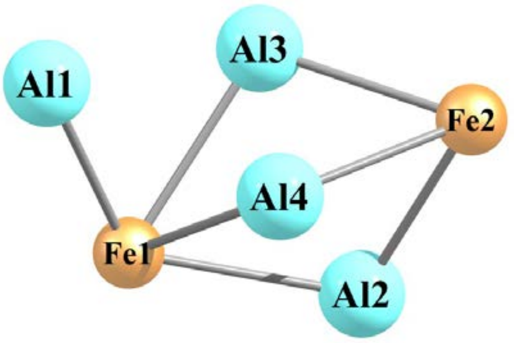

Al_4Fe_2 (5-X)

Molecular Structures
of Hexanuclear Al_3Fe_3 Metal Clusters
calculated by DFT OPBE/QZVP method

Al_3Fe_3 clusters having ground state with M_S= 2

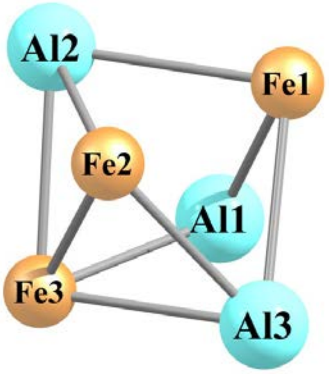

Al_3Fe_3 (2-I)

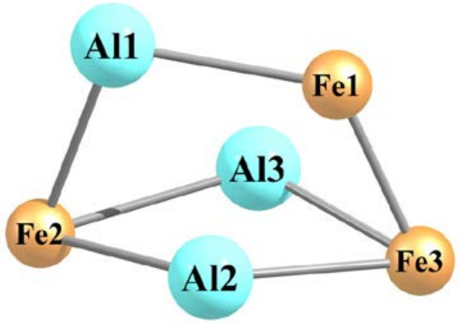

Al_3Fe_3 (2-II)

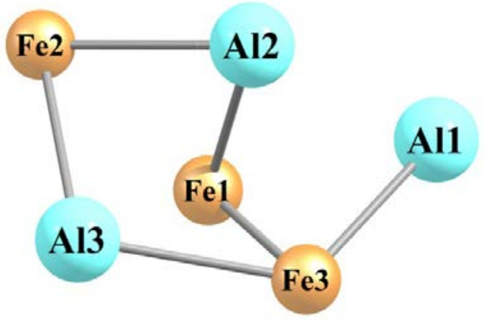

Al_3Fe_3 (2-III)

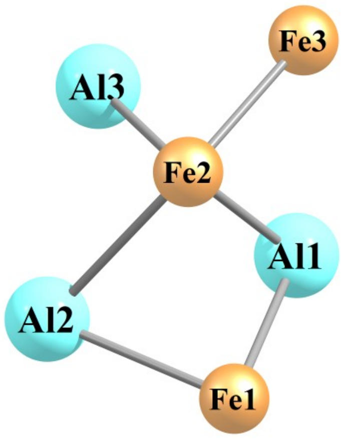

Al_3Fe_3 (2-IV)

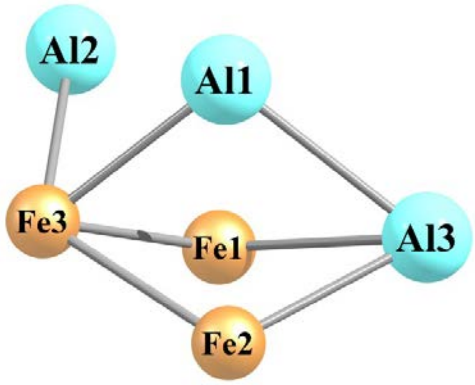

Al_3Fe_3 (2-V)

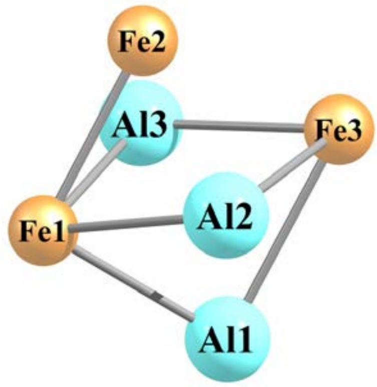

Al_3Fe_3 (2-VI)

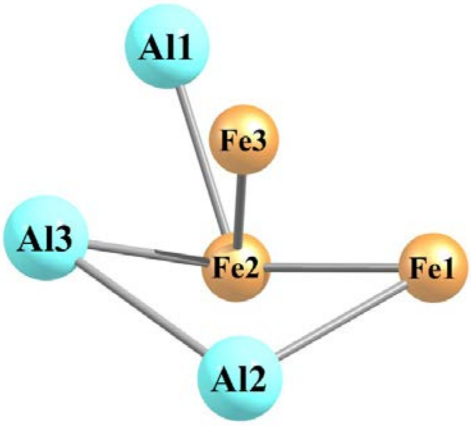

Al_3Fe_3 (2-VII)

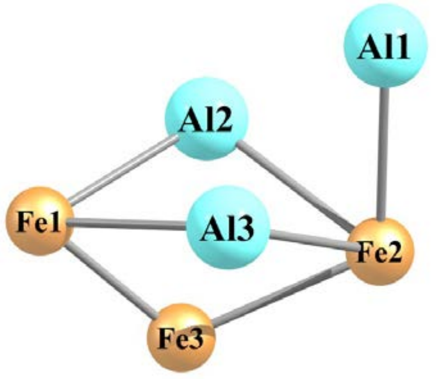

Al_3Fe_3 (2-VIII)

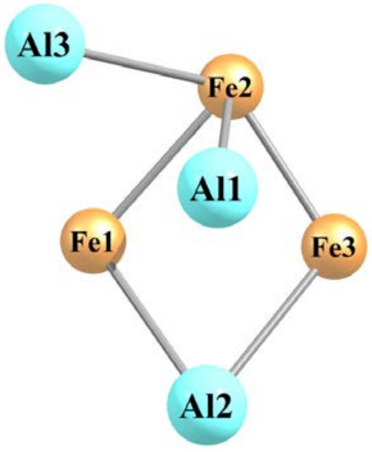

Al_3Fe_3 (2-IX)

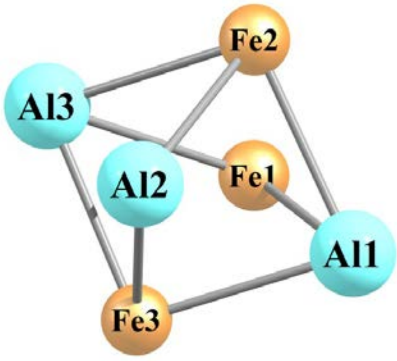

Al_3Fe_3 (2-X)

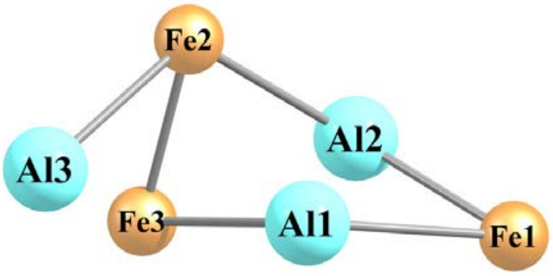

Al_3Fe_3 (2-XI)

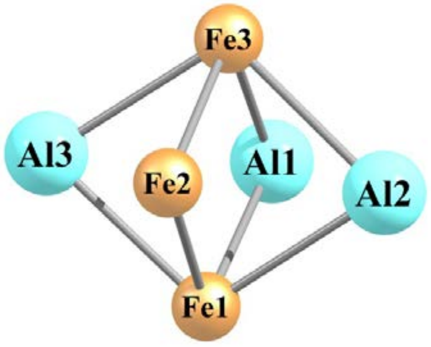

Al_3Fe_3 (2-XII)

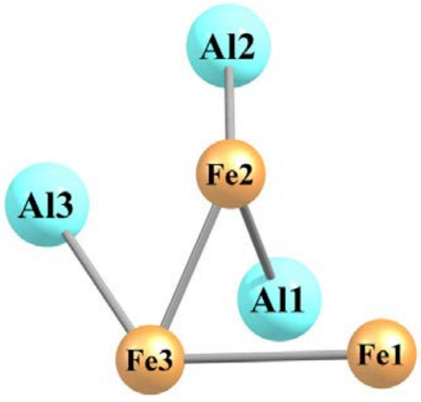

Al_3Fe_3 (2-XIII)

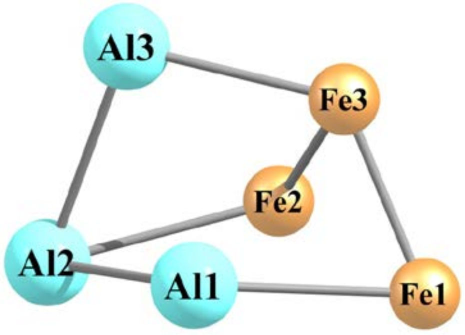

Al_3Fe_3 (2-XIV)

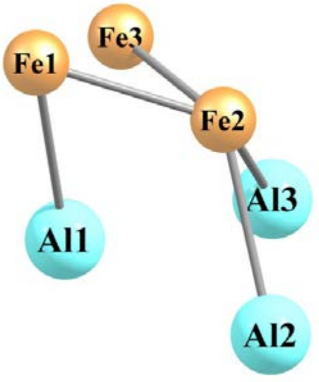

Al_3Fe_3 (2-XV)

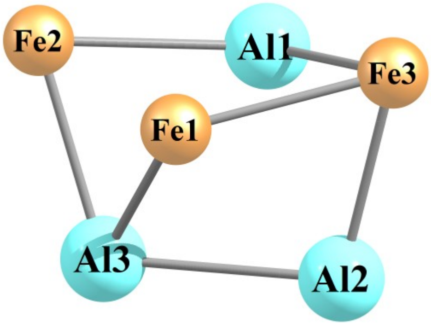

Al_3Fe_3 (2-XVI)

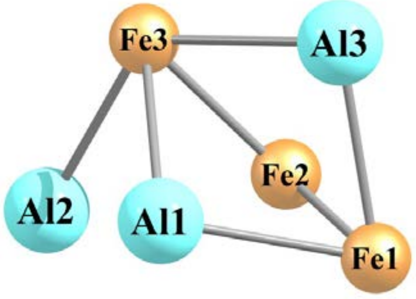

Al_3Fe_3 (2-XVII)

Al_3Fe_3 clusters having ground state with M_S= 4

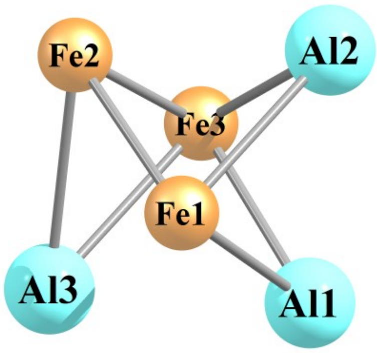

Al_3Fe_3 (4-I)

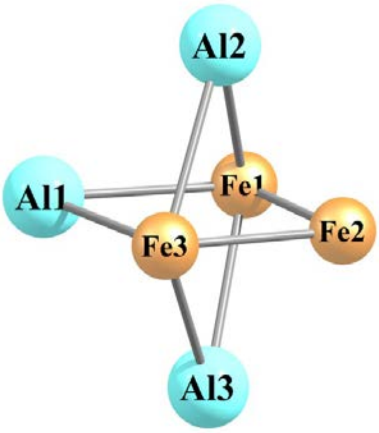

Al_3Fe_3 (4-II)

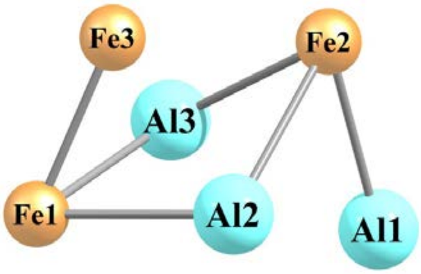

Al_3Fe_3 (4-III)

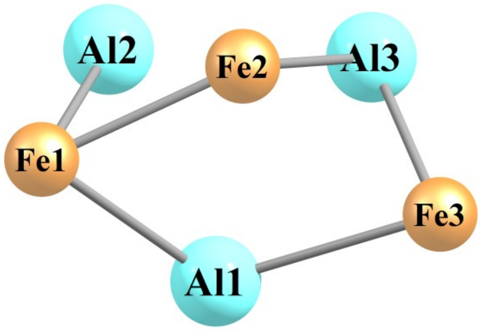

Al_3Fe_3 (4-IV)

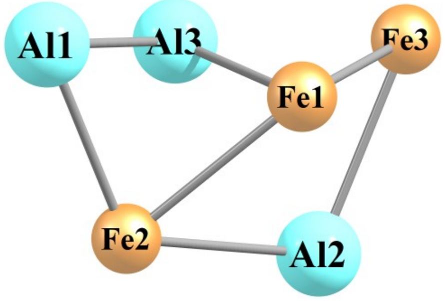

Al_3Fe_3 (4-V)

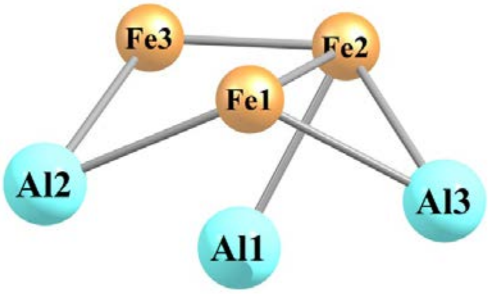

Al_3Fe_3 (4-VI)

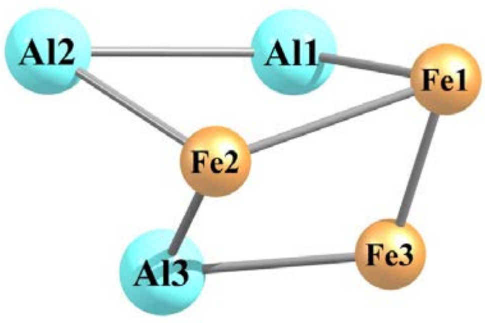

Al_3Fe_3 (4-VII)

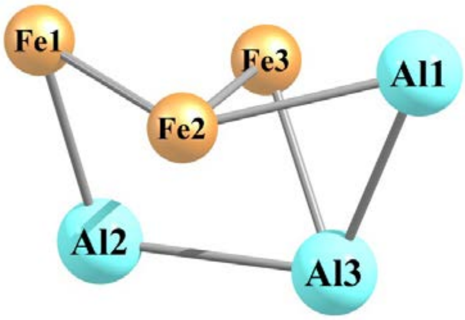

Al_3Fe_3 (4-VIII)

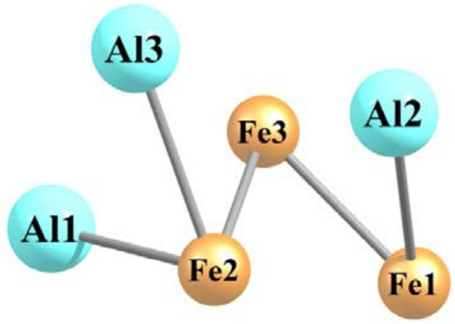

Al_3Fe_3 (4-IX)

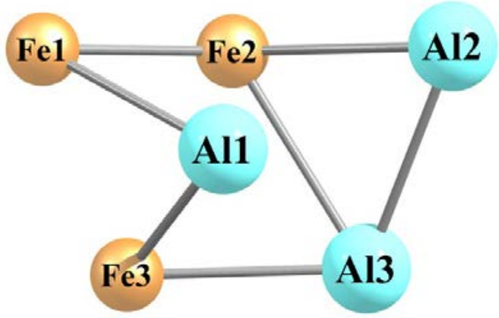

Al_3Fe_3 (4-X)

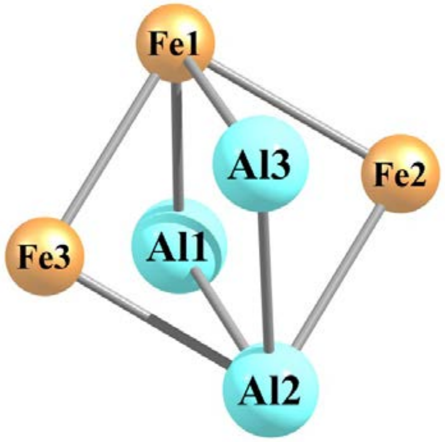

Al_3Fe_3 (4-XI)

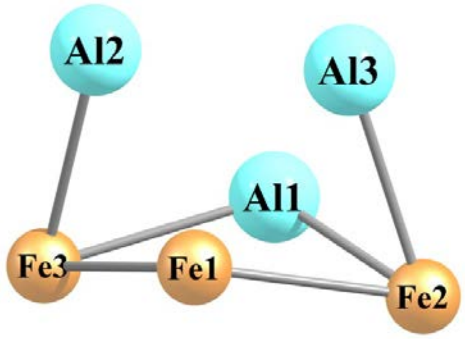

Al_3Fe_3 (4-XII)

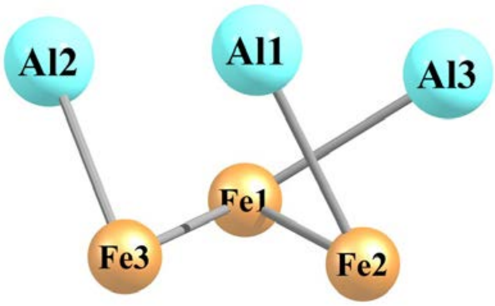

Al_3Fe_3 (4-XIII)

Al_3Fe_3 clusters having ground state with M_S= 6

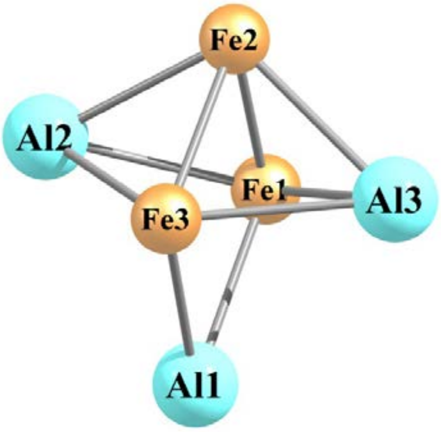

Al_3Fe_3 (6-I)

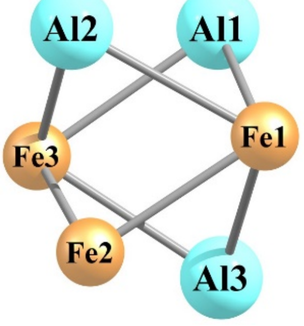

Al_3Fe_3 (6-II)

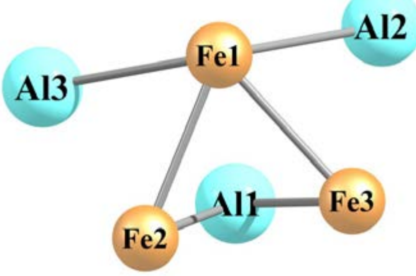

Al_3Fe_3 (6-III)

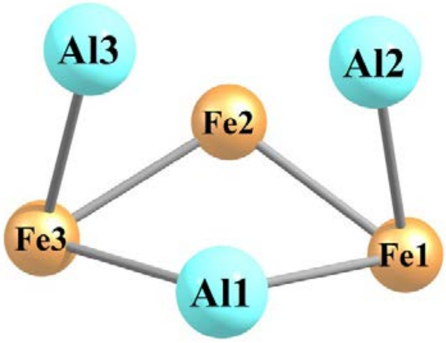

Al_3Fe_3 (6-IV)

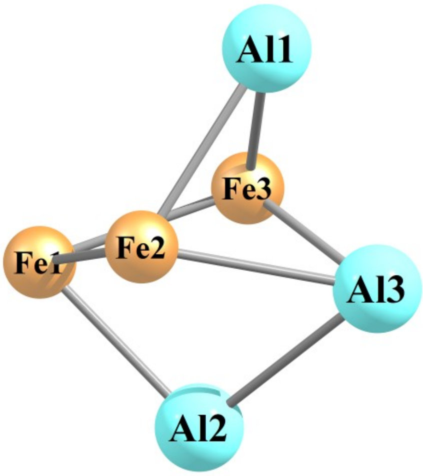

Al_3Fe_3 (6-V)

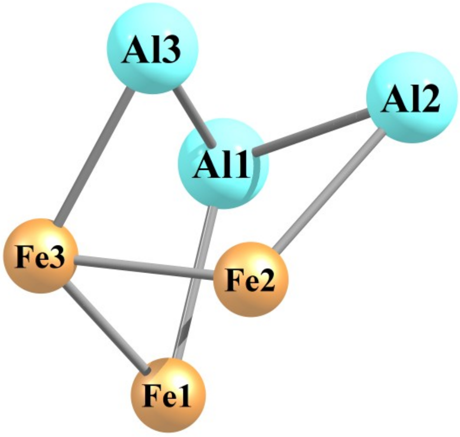

Al_3Fe_3 (6-VI)

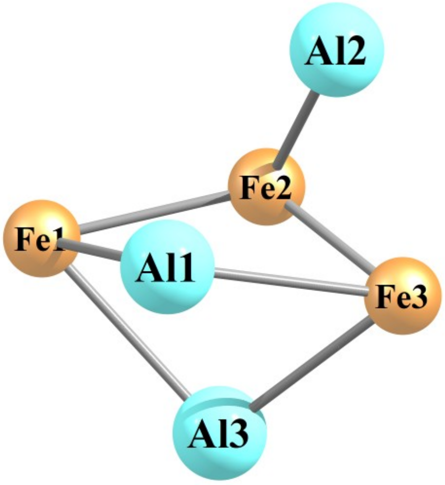

Al_3Fe_3 (6-VII)

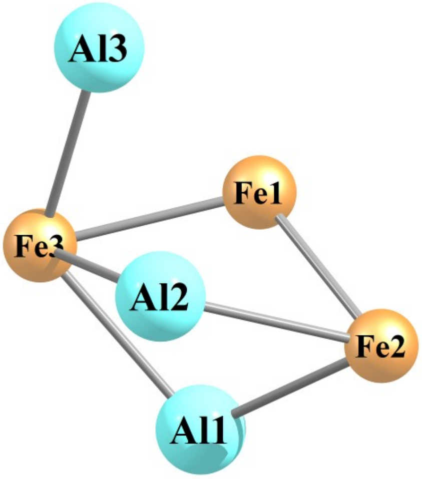

Al_3Fe_3 (6-VIII)

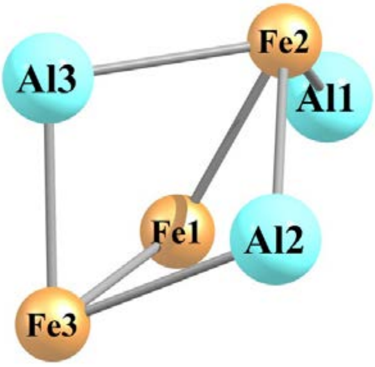

Al_3Fe_3 (6-IX)

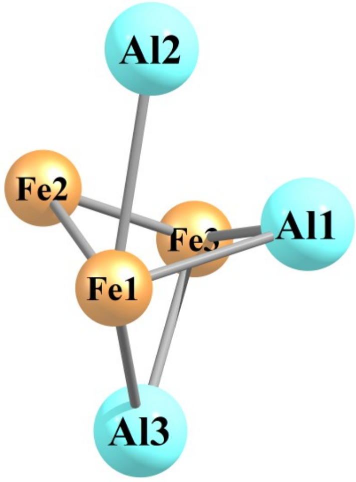

Al_3Fe_3 (6-X)

Molecular Structures of Hexanuclear Al_2Fe_4 Metal Clusters calculated by DFT OPBE/QZVP method

Al2Fe4 clusters having ground state with $M_S = 1$

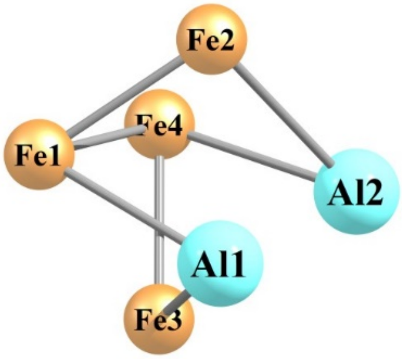

Al_2Fe_4 (1-I)

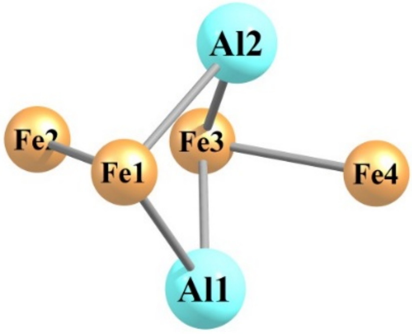

Al_2Fe_4 (1-II)

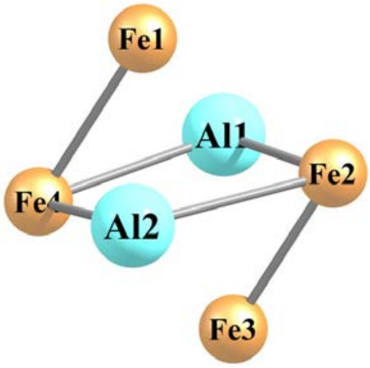

Al_2Fe_4 (1-III)

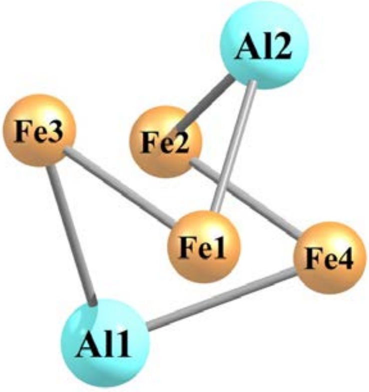

Al_2Fe_4 (1-IV)

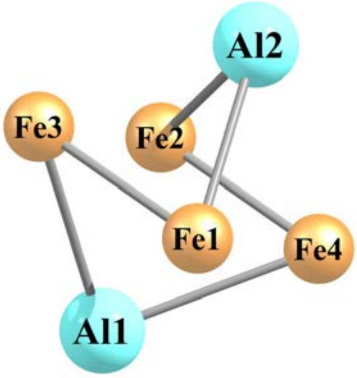

Al_2Fe_4 (1-V)

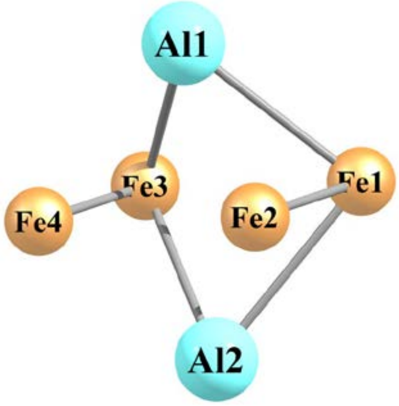

Al_2Fe_4 (1-VI)

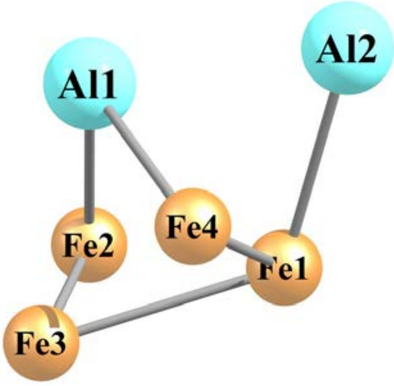

Al_2Fe_4 (1-VII)

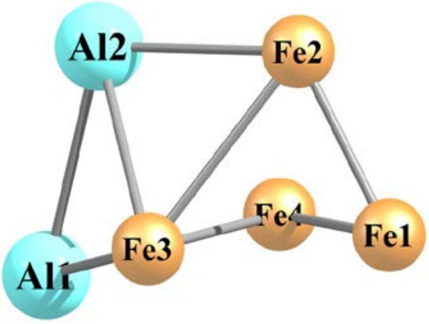

Al_2Fe_4 (1-VIII)

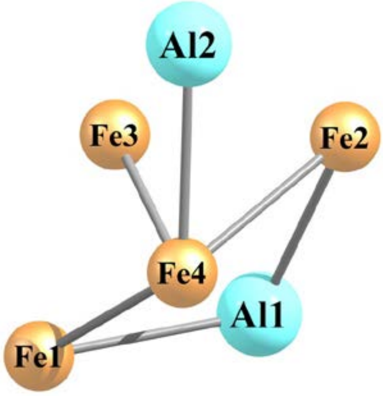

Al_2Fe_4 (1-IX)

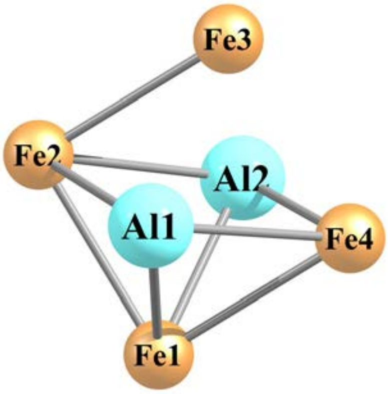

Al_2Fe_4 (1-X)

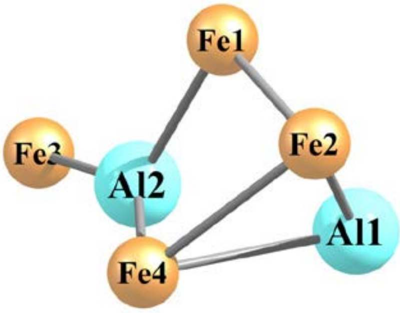

Al_2Fe_4 (1-XI)

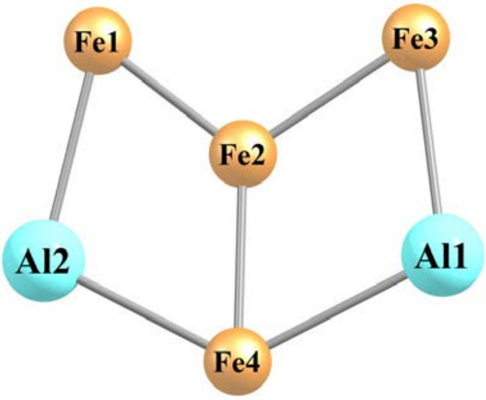

Al_2Fe_4 (1-XII)

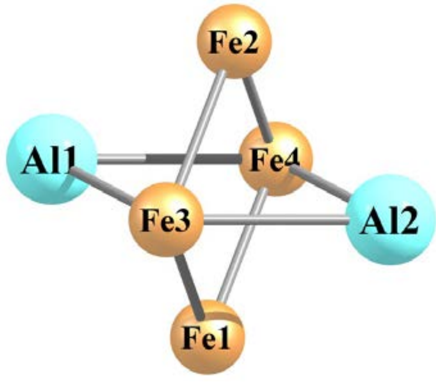

Al_2Fe_4 (1-XIII)

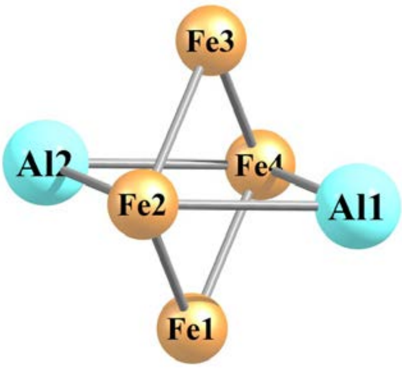

Al_2Fe_4 (1-XIV)

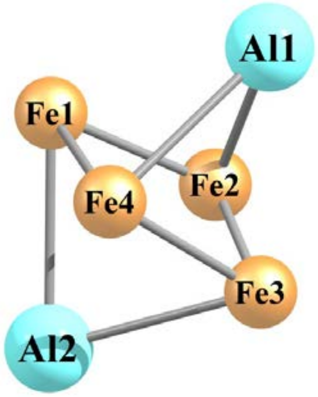

Al_2Fe_4 (1-XV)

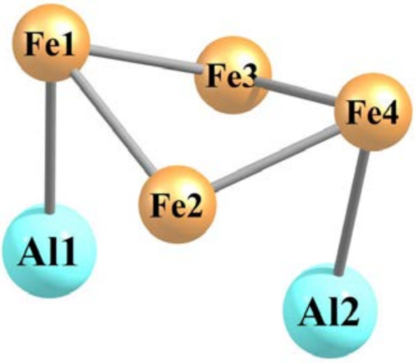

Al_2Fe_4 (1-XVI)

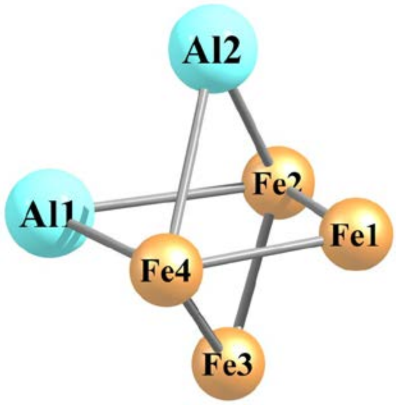

Al_2Fe_4 (1-XVII)

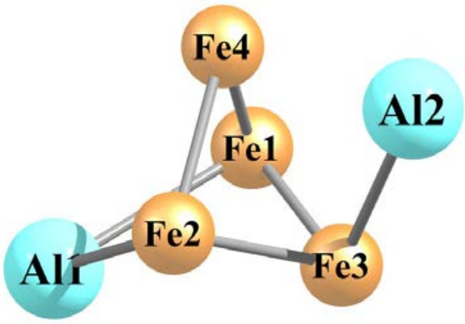

Al_2Fe_4 (1-XVIII)

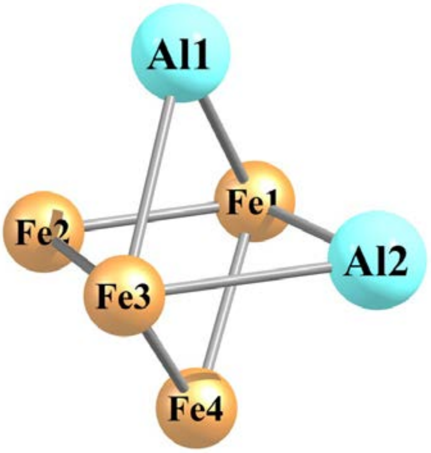

Al_2Fe_4 (1-XIX)

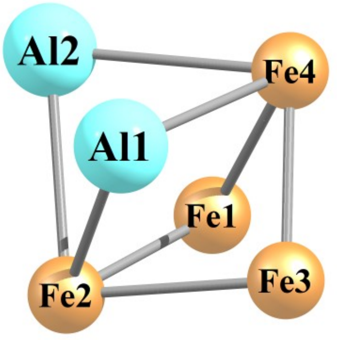

Al_2Fe_4 (1-XX)

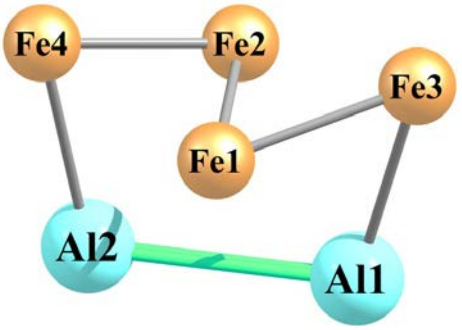

Al_2Fe_4 (1-XXI)

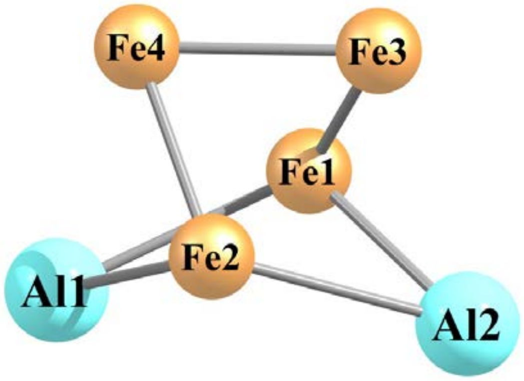

Al_2Fe_4 (1-XXII)

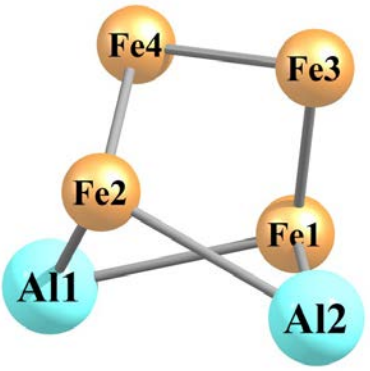

Al_2Fe_4 (1-XXIII)

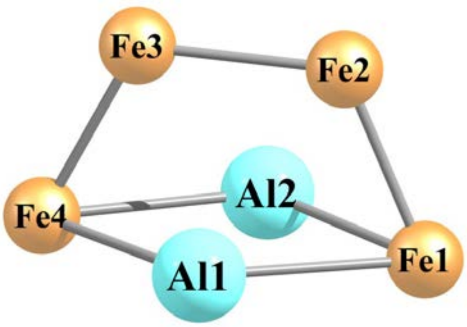

Al_2Fe_4 (1-XXIV)

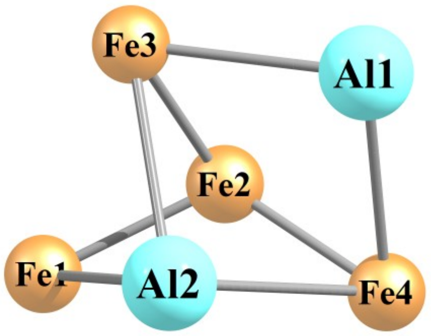

Al$_2$Fe$_4$ (1-XXV)

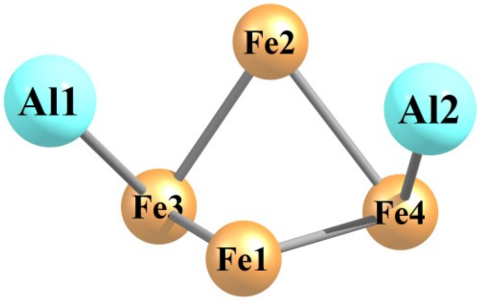

Al$_2$Fe$_4$ (1-XXVI)

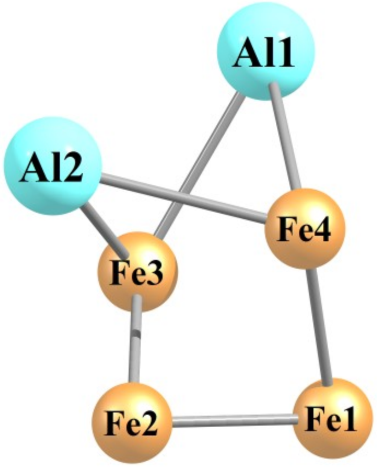

Al$_2$Fe$_4$ (1-XXVII)

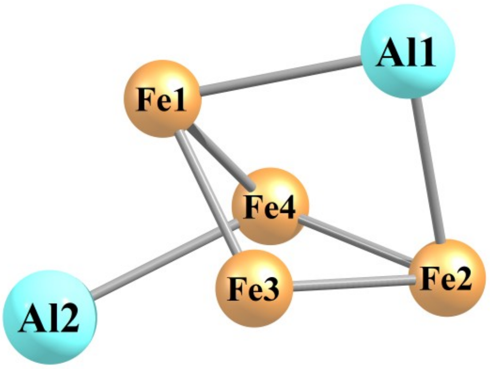

Al$_2$Fe$_4$ (1-XXVIII)

Al$_2$Fe$_4$ clusters having ground state with M_S= 3

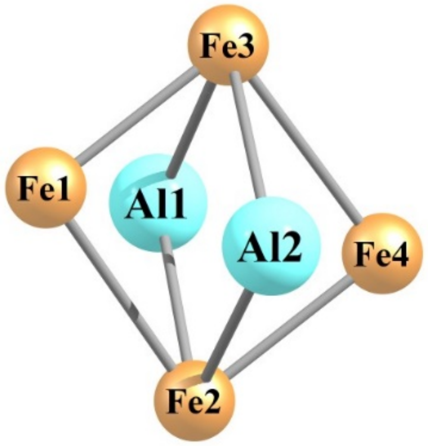

Al$_2$Fe$_4$ (3-I)

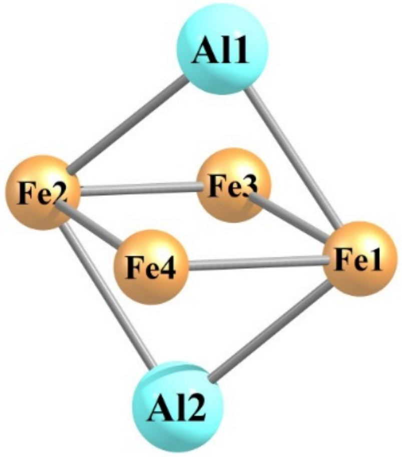

Al$_2$Fe$_4$ (3-II)

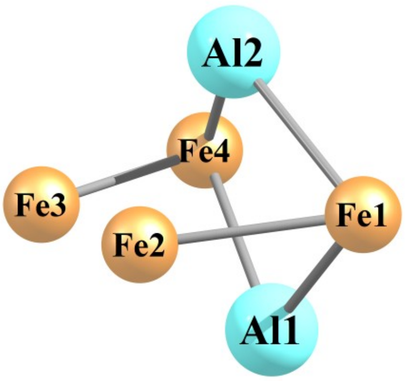

Al$_2$Fe$_4$ (3-III)

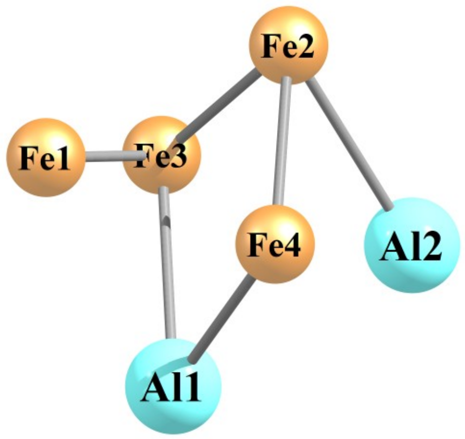

Al_2Fe_4 (3-IV)

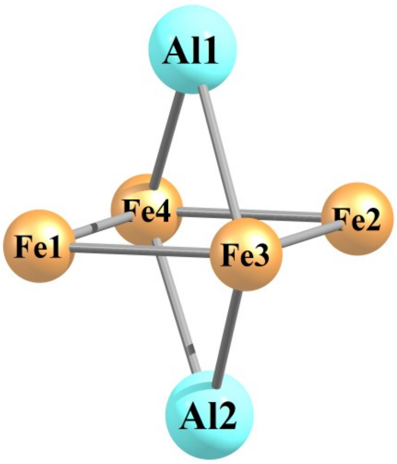

Al_2Fe_4 (3-V)

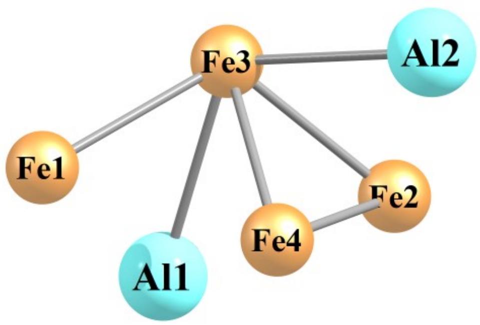

Al_2Fe_4 (3-VI)

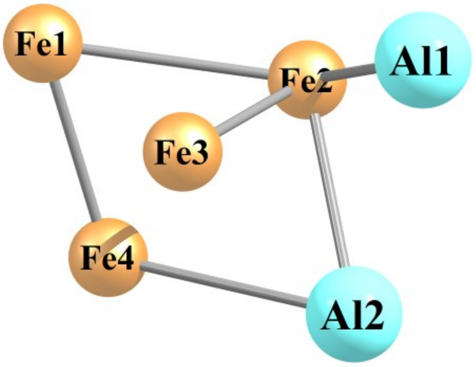

Al_2Fe_4 (3-VII)

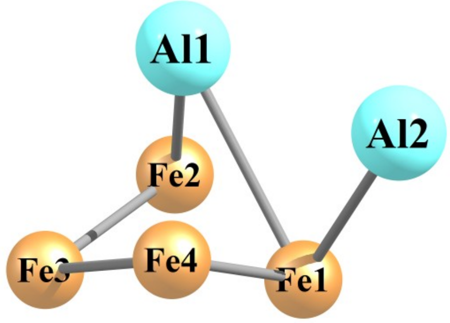

Al_2Fe_4 (3-VIII)

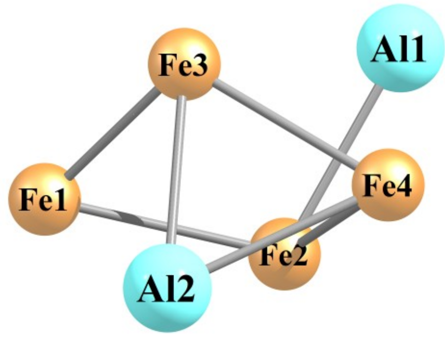

Al_2Fe_4 (3-IX)

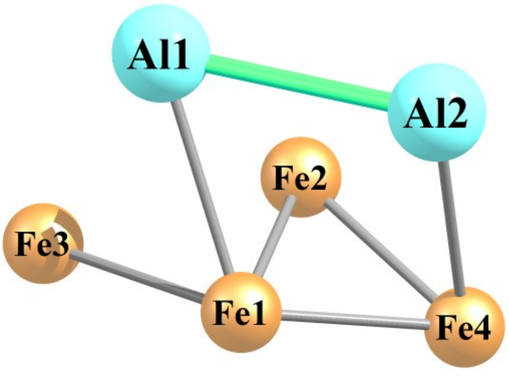

Al_2Fe_4 (3-X)

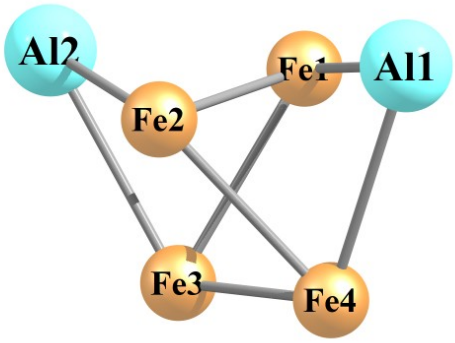

Al_2Fe_4 (3-XI)

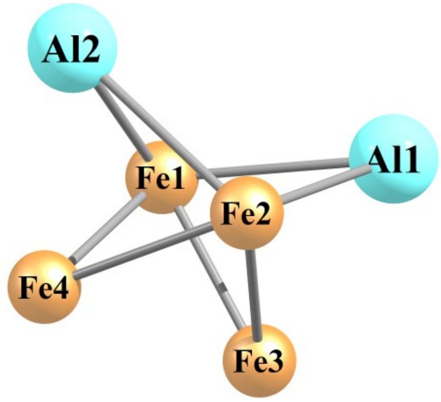

Al_2Fe_4 (3-XII)

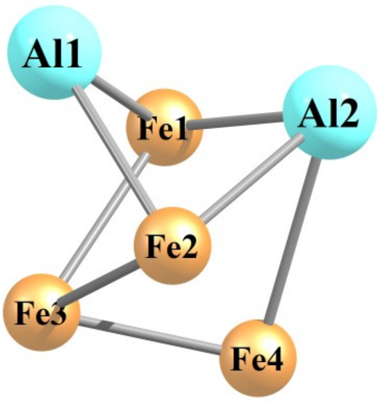

Al_2Fe_4 (3-XIII)

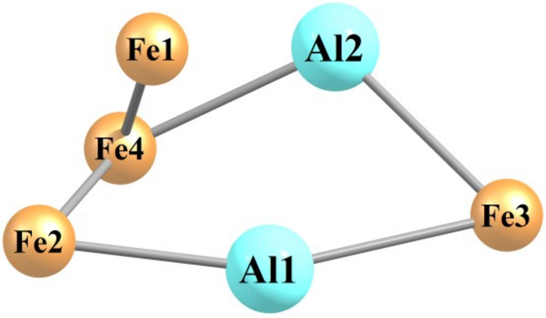

Al_2Fe_4 (3-XIV)

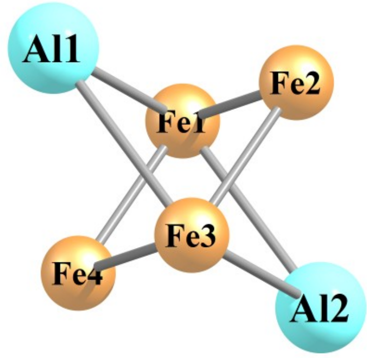

Al_2Fe_4 (3-XV)

Al_2Fe_4 clusters having ground state with $M_S = 5$

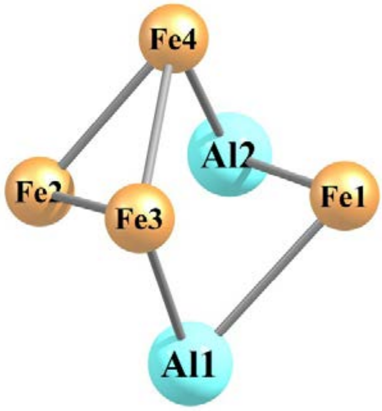

Al_2Fe_4 (5-I)

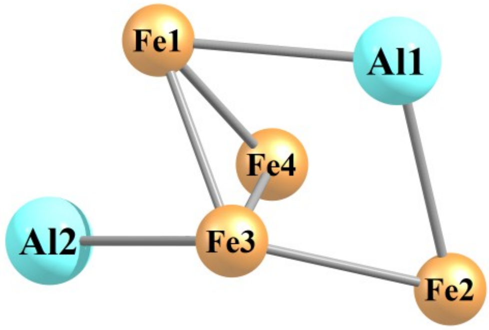

Al_2Fe_4 (5-II)

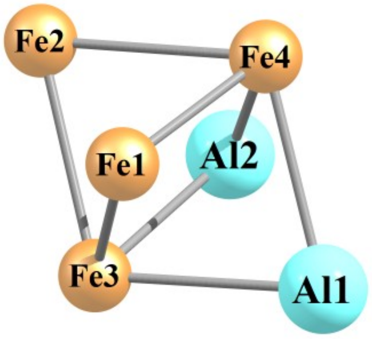

Al_2Fe_4 (5-III)

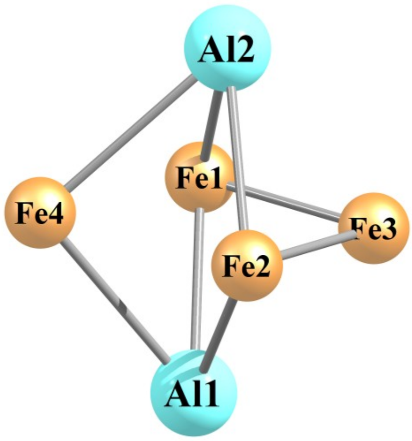

Al_2Fe_4 (5-IV)

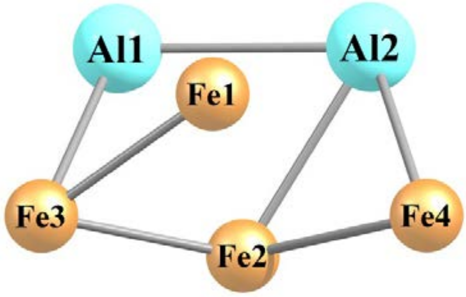

Al_2Fe_4 (5-V)

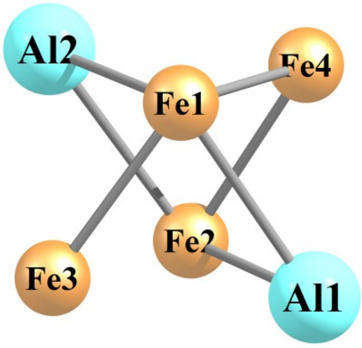

Al_2Fe_4 (5-VI)

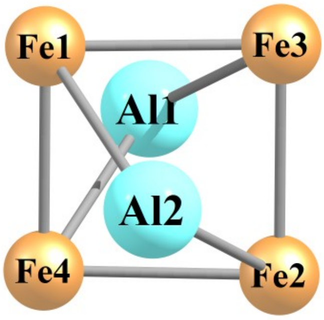

Al_2Fe_4 (5-VII)

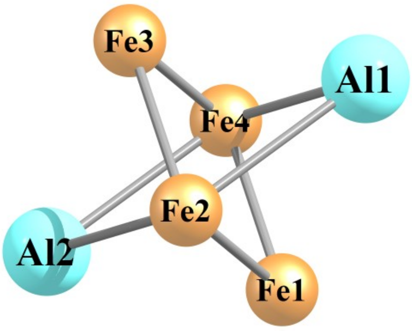

Al_2Fe_4 (5-VIII)

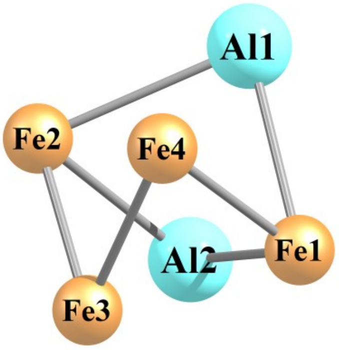

Al_2Fe_4 (5-IX)

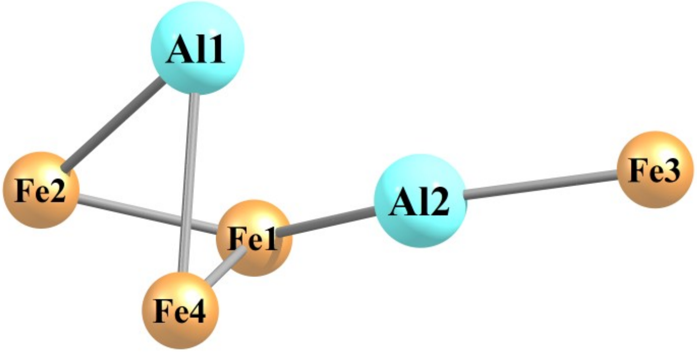

Al_2Fe_4 (5-X)

Molecular Structures of Hexanuclear $AlFe_5$ Metal Clusters calculated by DFT OPBE/QZVP method

$AlFe_5$ clusters having ground state with $M_S = 2$

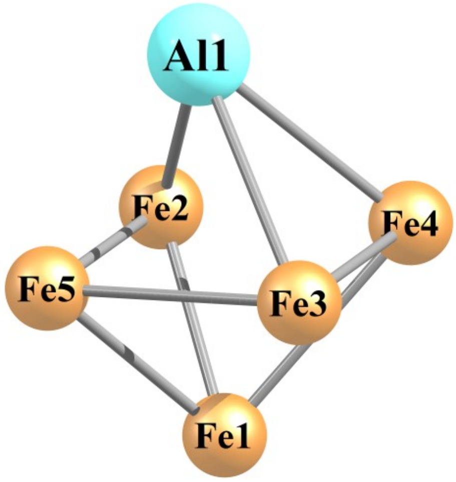

$AlFe_5$ (2-I)

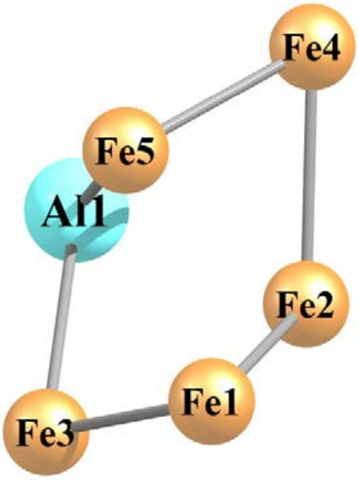

$AlFe_5$ (2-II)

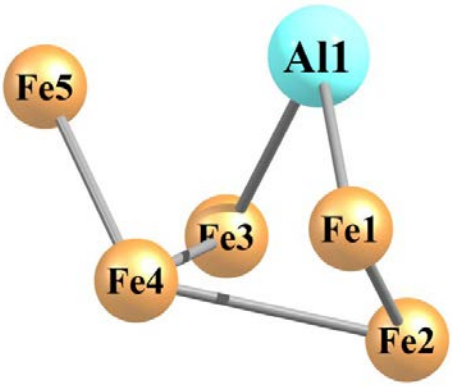

$AlFe_5$ (2-III)

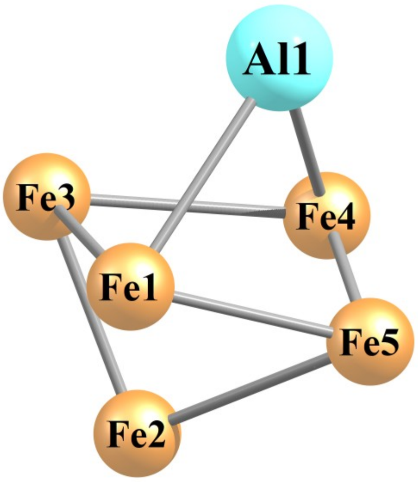

$AlFe_5$ (2-IV)

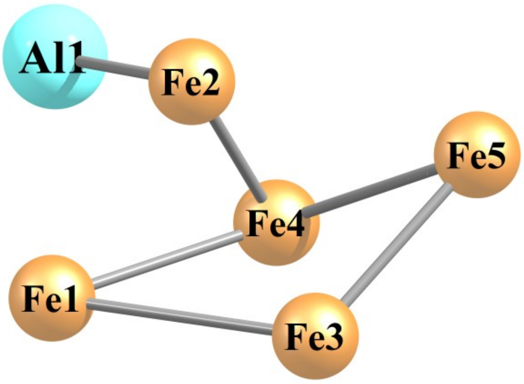

$AlFe_5$ (2-V)

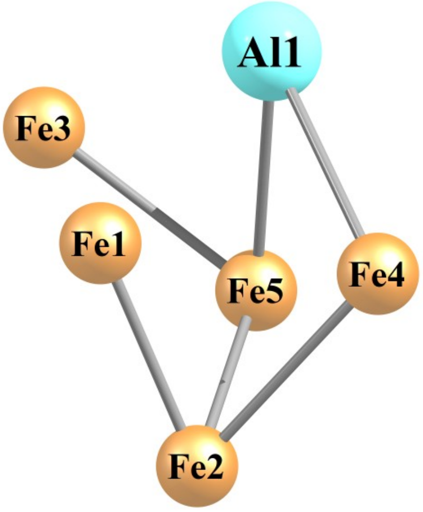

$AlFe_5$ (2-VI)

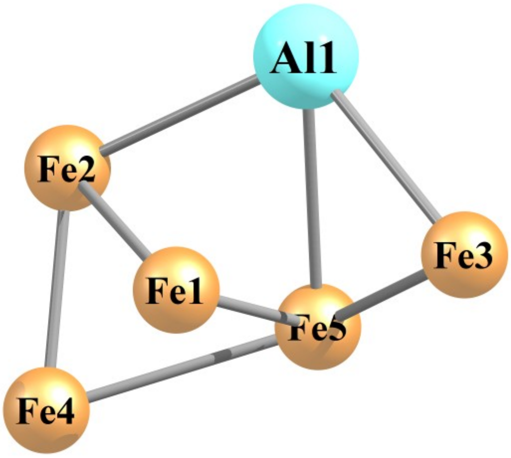

$AlFe_5$ (2-VII)

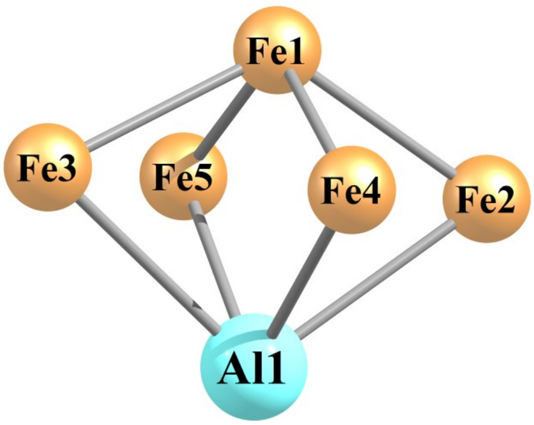

$AlFe_5$ (2-VIII)

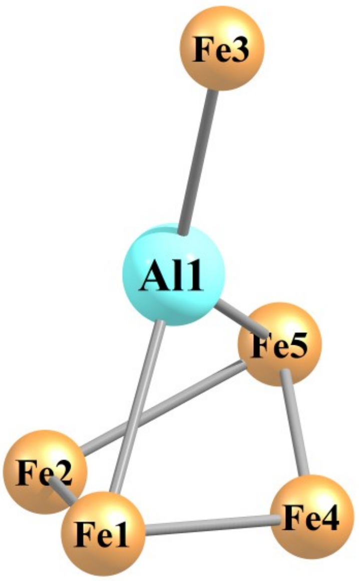

$AlFe_5$ (2-IX)

AlFe$_5$ clusters having ground state with M_S= 4

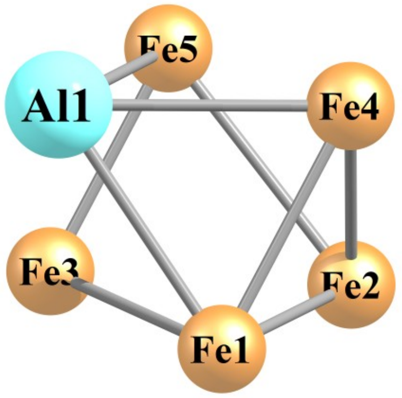

AlFe$_5$ (4-I)

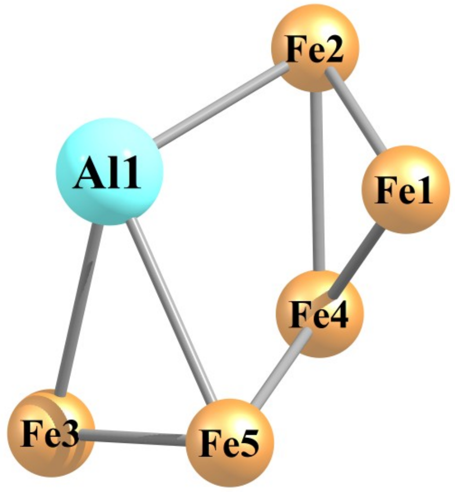

AlFe$_5$ (4-II)

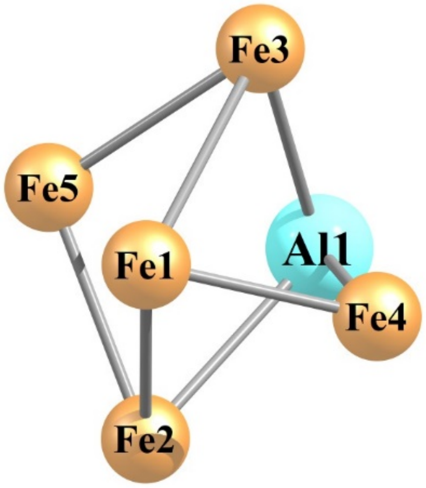

AlFe$_5$ (4-III)

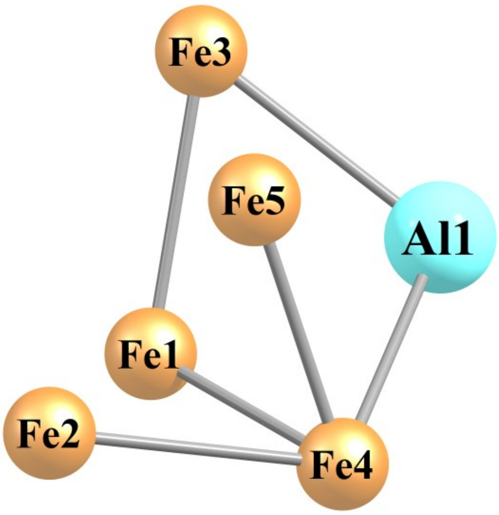

AlFe$_5$ (4-IV)

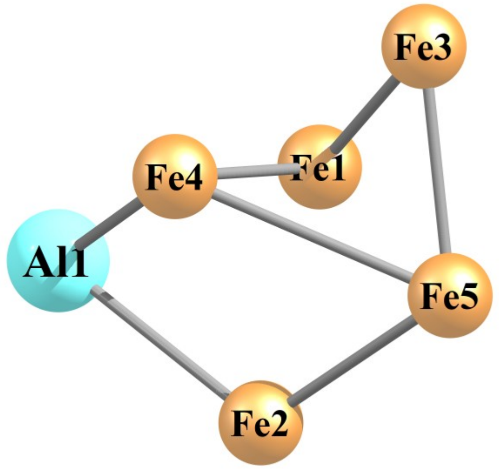

AlFe$_5$ (4-V)

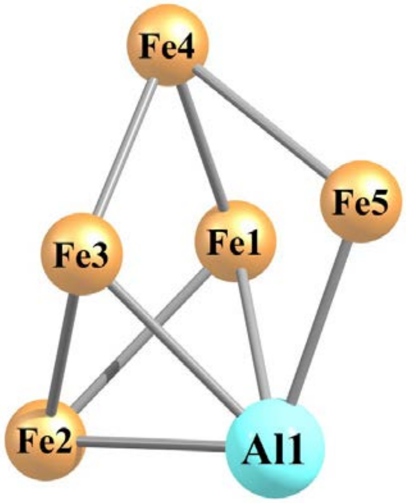

AlFe$_5$ (4-VI)

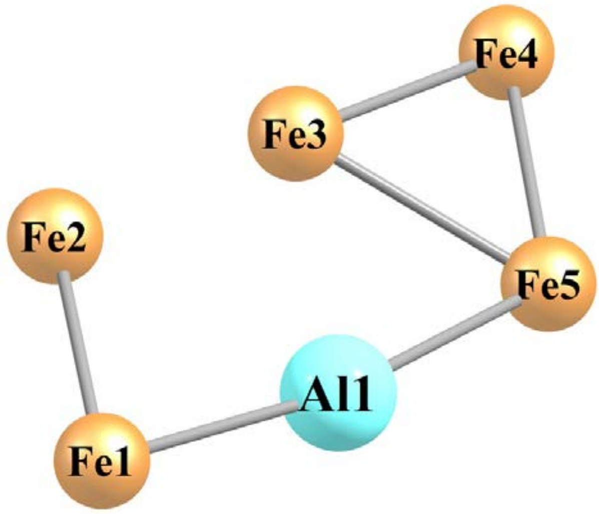

AlFe$_5$ (4-VII)

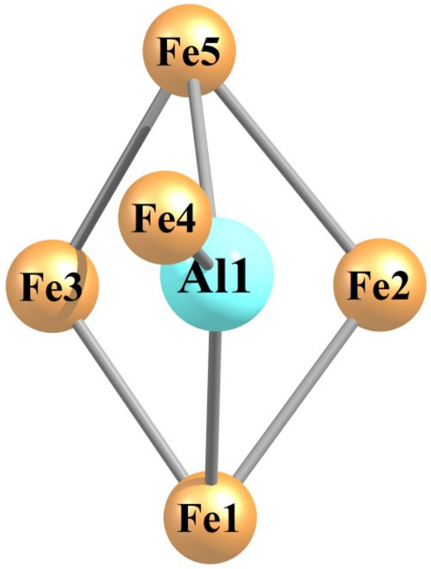

AlFe$_5$ (4-VIII)

AlFe$_5$ clusters having ground state with M_S= 6

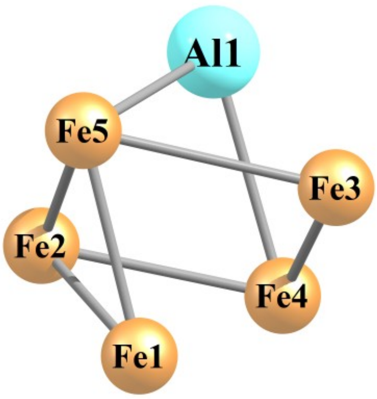

AlFe$_5$ (6-I)

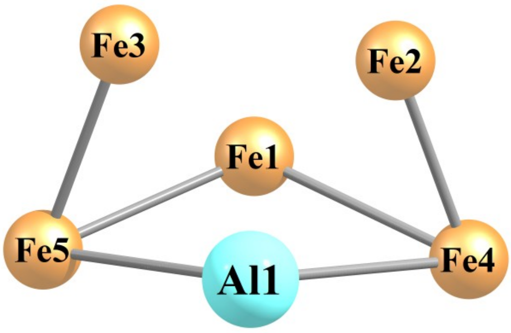

AlFe$_5$ (6-II)

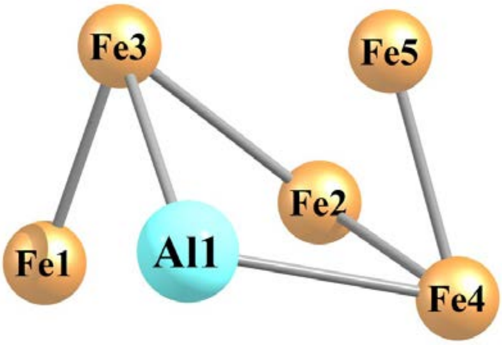

AlFe$_5$ (6-III)

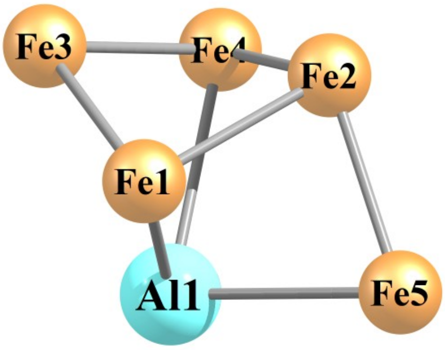

AlFe$_5$ (6-IV)

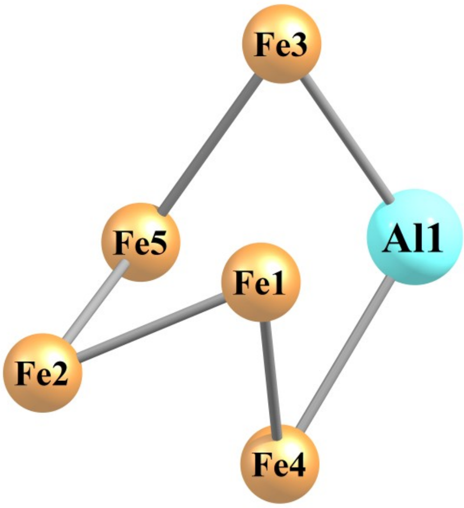

AlFe$_5$ (6-V)

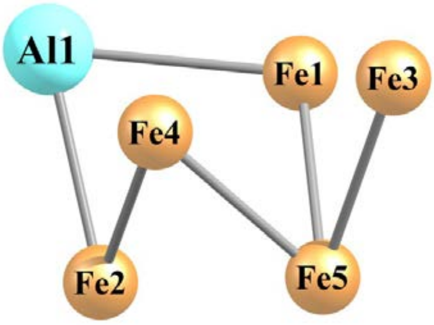

AlFe$_5$ (6-VI)
